# Microbiologically influenced corrosion—more than just microorganisms

**DOI:** 10.1093/femsre/fuad041

**Published:** 2023-07-12

**Authors:** J Knisz, R Eckert, L M Gieg, A Koerdt, J S Lee, E R Silva, T L Skovhus, B A An Stepec, S A Wade

**Affiliations:** Department of Water Supply and Sewerage, Faculty of Water Sciences, University of Public Service, 6500, Baja, Hungary; Microbial Corrosion Consulting, LLC, Commerce Township, 48382, MI, USA; Petroleum Microbiology Research Group, Department of Biological Sciences, University of Calgary, 2500 University Drive NW, Calgary, Alberta T2N 1N4, Canada; Federal Institute for Materials Research and Testing (BAM), 12205, Berlin, Germany; Naval Research Laboratory, Ocean Sciences Division, Stennis Space Center, 39529, MS, USA; BioISI—Biosystems and Integrative Sciences Institute, Faculty of Sciences, University of Lisboa, Campo Grande, C8 bdg, 1749-016, Lisboa, Portugal; CERENA - Centre for Natural Resources and the Environment, Instituto Superior Técnico, University of Lisbon, Av. Rovisco Pais, 1, 1049-001, Lisboa, Portugal; Research Center for Built Environment, Energy, Water and Climate, VIA, University College, 8700, Horsens, Denmark; Department of Energy and Technology, NORCE Norwegian Research Centre AS, Nygårdsgaten 112, 5008 Bergen, Norway; Bioengineering Research Group, Swinburne University of Technology, 3122, Melbourne, Australia

**Keywords:** microbiologically influenced corrosion, biocorrosion, biodeterioration, interdisciplinarity, multiple lines of evidence

## Abstract

Microbiologically influenced corrosion (MIC) is a phenomenon of increasing concern that affects various materials and sectors of society. MIC describes the effects, often negative, that a material can experience due to the presence of microorganisms. Unfortunately, although several research groups and industrial actors worldwide have already addressed MIC, discussions are fragmented, while information sharing and willingness to reach out to other disciplines are limited. A truly interdisciplinary approach, which would be logical for this material/biology/chemistry-related challenge, is rarely taken. In this review, we highlight critical non-biological aspects of MIC that can sometimes be overlooked by microbiologists working on MIC but are highly relevant for an overall understanding of this phenomenon. Here, we identify gaps, methods, and approaches to help solve MIC-related challenges, with an emphasis on the MIC of metals. We also discuss the application of existing tools and approaches for managing MIC and propose ideas to promote an improved understanding of MIC. Furthermore, we highlight areas where the insights and expertise of microbiologists are needed to help progress this field.

## Teaser

The purpose of this review is to provide microbiologists with background information on the non-microbial aspects of microbiologically influenced corrosion (MIC) to directly tackle the siloed nature of MIC research and encourage them to work on this important topic.

## Introduction

Engineered materials are essential to our society to ensure our current prosperity and sustainability in the future. A wide range of matters such as energy supply, food, transportation, housing, and various other of life’s fundamentals rely upon engineered materials. To support this, a variety of materials are used that can have limited lifetimes; thus, repair or replacement is inevitable in the long run. Very often, however, the expected lifetimes of materials are not achieved. In many cases, damage occurs much earlier than expected, causing costly production downtime and repair expenses, and in some cases, even major environmental problems. More often than thought, microorganisms play a major role in this damage, and microbiologists are key to help improve our understanding of the associated problems as well as potential solutions.

It is scientifically proven that many metallic and non-metallic materials (e.g. concrete, wood, and plastic) can be deteriorated by microorganisms, with detrimental (e.g. asset failure) or beneficial (e.g. biodegradation of plastic) consequences. However, biodeterioration of metals in our built environment can result in significant issues of production loss, environmental disasters, and/or asset safety (Jacobson 2007). While the topic of microbiologically influenced corrosion (MIC) has been known and studied for decades, its understanding is limited, as are the methods for prevention and monitoring, and it poses great challenges in many industrial settings. Fundamentally, a collaborative effort from various scientific and technical disciplines, including microbiology, material science, process and electrochemistry, biochemistry, corrosion engineering, and integrity management, is needed to help progress the understanding of MIC. This combination of knowledge is critical to determine the root causes of a failure associated with MIC and to develop effective long-term mitigation and monitoring strategies specifically adapted to the associated system (Silva et al. 2021, Eckert and Skovhus 2022).

In recent decades, our understanding about the microorganisms, the mechanisms, and the factors related to MIC has grown enormously, yet many questions remain unanswered. In general, development in this field has been slowed due to poor communications between academia and industry, as well as amongst different disciplines within academia working on corrosion (abiotic/biotic). Unjustifiably, MIC is still considered a questionable mechanism in many industrial sectors, and in some cases, its existence is even denied, hindering important knowledge transfer and the development of environmentally appropriate solutions for this problem. In academia, some microbiologists working on MIC can be hampered by limited access to, or knowledge of, important aspects of abiotic corrosion or methods used by engineers and materials scientists. The lack of information exchange between industry and academy means that scientists are not aware of the actual needs of the industry, thus academic research can lack practical relevance.

A key historical problem with MIC studies is the often-siloed nature of scientific disciplines working on the topic. While the number of MIC-related research articles has grown significantly in the past two decades, many of these articles only cover one or two aspects of this multidisciplinary topic. For example, Hashemi et al. (2017) demonstrated that a large proportion of the research published on MIC was siloed separately within the corrosion/materials science and microbiology areas, despite the multidisciplinary nature of MIC. With such siloing of knowledge, valuable information can become isolated within one particular discipline instead of spreading amongst the wider MIC community. This consequently delays progress and innovation, despite the huge economic and environmental impact of MIC.

The focus of the current review is to directly tackle one aspect, the siloed nature of MIC research, by providing important background information on the non-microbiological aspects of MIC to microbiologists. While microbiologists are experts in their fields, they typically have limited understanding of the corrosion/metallurgical and/or chemical aspects of MIC. This review, which has an emphasis on the degradation of metals, will provide information to help avoid mistakes that can be made when experts in one field start working on a multidisciplinary topic such as MIC. By providing this information, we aim to encourage interdisciplinary and intersectoral collaborations to ease the entrance of biological scientists, including microbiologists, into the complex field of MIC and, ultimately, to shape the next era of MIC research and management.

## MIC mechanisms and clarification of terminology

MIC has been defined by NACE and ASTM as “corrosion affected by the presence or activity, or both, of microorganisms” (ASTM G193 2022), as adapted from Little and Lee (2007). Several terms are used to describe this phenomenon (microbially influenced corrosion, MIC, biodeterioration, and biocorrosion), with the range of different terms often resulting in confusion. Here we aim to clarify some of the key terminology used in relation to the phenomenon itself or to its various mechanisms.

### MIC terminology

A broader term that defines the microbial degradation of metallic and non-metallic materials is *biodeterioration*, i.e. “any undesirable change in the properties of a material caused by the vital activities of organisms” (Hueck 1965). The fundamental cycling processes involved in the biodeterioration of stone and metal have recently been reviewed by Gaylarde and Little (2022). *MIC* is commonly associated with the biodeterioration of materials such as metals and concrete. In Europe and in some international standards, the term *corrosion* is used only for metallic material (ISO 8044-2020), but the International Union of Pure and Applied Chemistry (IUPAC) (1997) provides a broader and widely accepted definition, i.e. “corrosion is an irreversible interfacial reaction of a material (metal, ceramic, or polymer) with its environment that results in consumption of the material or in the dissolution into the material of a component of the environment.”

The term *biocorrosion* is increasingly used as a synonym of MIC, although this term can create confusion as, in the US, it primarily refers to the corrosion of medical implants due to both biotic and abiotic processes (Little et al. 2020b). It has been suggested that the term *microbial corrosion* hints that microorganisms are the main cause of the corrosion (Gu 2012), while others use this term as a synonym for MIC. The ISO 8044 standard describes the term *bacterial corrosion* as a synonym for MIC if it is solely due to the activity of bacteria; however, as archaea or even fungi can be involved in the deterioration process, this term should only be used in unequivocal cases.

*MIC* is probably the most widely used term to describe the many ways in which microorganisms can affect corrosion processes. While some use the word *induced* instead of *influenced*, the presence/activity of certain microorganisms has also been known to reduce the rates of corrosion (Videla and Herrera 2009, Kip and Van Veen 2015) and so, the term *induced* is not as broadly applicable. The adjective “microbiologically” in the term of MIC is grammatically incorrect as it refers to corrosion caused by microbiology instead of microorganisms; nonetheless, at the CORROSION/90 conference in Las Vegas, Nevada, NACE’s Publication Committee supported its use for future NACE documents (Brooke 1990). Since then, many other associations and standards have adopted the term *MIC*, e.g. (GRI 1990, AMPP 2018); thus, we prefer to use this term in this review to align with these standards. However, readers are free to decide their preference, as MIC can be the acronym for both microbiologically and microbially influenced corrosion as well as for microbial corrosion, and all three terms are suitable to name the phenomenon. If one prefers, *biocorrosion* can also be used as long as it is properly defined what users mean under the term.

*Biofouling* can lead to MIC, but the term cannot be used as a synonym for MIC. Biofouling is the accumulation and growth of various organisms, including microorganisms (microfouling), plants, and/or animals (e.g. algae, barnacles) (macrofouling), on a surface (AMPP 2023). While, in some cases, biofouling can be associated with corrosion, this is not always the case. Indeed, other problems associated with biofouling, including increased water resistance on ships, increased fuel consumption due to drag (Callow and Callow 2011, Tulcidas et al. 2015), or the introduction of non-indigenous species (Li and Ning 2019), can be more of an issue. Thus, using biofouling as a direct synonym for MIC is not recommended.

### MIC mechanisms of metals

The term MIC does not describe a single mechanism for corrosion, it is rather a collective term for a variety of different mechanisms through which microorganisms alter the kinetics of corrosion reactions by their presence or activity (Lee et al. 2022). For MIC to occur, the specific interplay of the “three M’s” is required: microorganisms, media, and metals (Little et al. 2020b). The combination of these interactions defines the various mechanisms that can change the rate of metal deterioration, either directly or indirectly. There have been many reviews specifically describing MIC mechanisms, but unfortunately, there are many inconsistencies with the terminologies used, making it difficult to navigate among them. Here we aim to clarify the terminology used for MIC mechanisms without going very deep into details, with the goal of providing a common and easily understood language.

### MIC due to surface deposition

Microorganisms are involved in a range of processes that can lead to the formation of deposits on the surfaces of materials. They can form single or multispecies communities attached to a surface, known as biofilms, which are often embedded in a self-produced matrix of extracellular polymeric substances (EPS) (Flemming et al. 2016). Alternatively, metabolic processes due to some types of microorganisms, such as metal-oxidizing bacteria, can lead to metal products being deposited on a surface (Lee and Little 2019). As discussed below, these deposits can affect and, in some cases, accelerate corrosion.

MIC mechanisms are often classified based on oxygen presence and/or availability in a given environment. However, in real-life conditions, oxygen may intermittently be available and/or consumed by microorganisms that can react directly with the metal surface. Thus, instead of a strictly aerobic or anaerobic environment, an oxygen gradient is often present that can vary over time. This clashes somewhat with many laboratory-based MIC experiments, which aim to operate under strictly aerobic or anaerobic conditions. Indeed, there is potential scope for more experiments to be performed that look at the effect of alternating oxygen in MIC tests. In one example, Lee et al. (2004) showed that SRB corrosion rates increased by a factor of three if oxygen was intermittently present, when compared to either strictly aerobic or anaerobic conditions.

The growth of a biofilm itself can result in MIC in aerobic fluid environments when biofilms are formed in a patchy arrangement, creating **oxygen concentration** or **differential aeration cells** between the anodic and the cathodic areas of the surface. Fundamentally, the mechanism of corrosion involves electron flow through the metal from the anode to the cathode, where (under aerobic conditions) oxygen is the electron acceptor (Hamilton 2003). The biofilm, which defines the anode, prevents oxygen reaching the metal surface while the metabolism of aerobic bacteria uses up the oxygen present in the biofilm. The cathodic site ends up being the area uncovered by the biofilm that is exposed to oxygen. Roe et al. (1996) have shown that cell-free EPS alone can initiate corrosion.

Other surface deposits, such as metal oxides, can form oxygen concentration cells, resulting in **under deposit corrosion** or **oxygen gradient corrosion**. The two most studied groups of MIC-related aerobic metal-depositing bacteria are iron-oxidizing bacteria (FeOB) and manganese-oxidizing bacteria (MnOB), which have been reviewed by Lee and Little (2019). For example, FeOB oxidize Fe^2+^ into Fe^3+^ in an oxygen-rich environment where the area underneath the accumulated iron oxides is depleted of oxygen and a small anode is formed relative to the surrounding large, oxygen-saturated cathode. The difference in dissolved oxygen concentration creates a potential difference resulting in oxygen concentration cells or, alternatively, can cause a form of galvanic corrosion. These deposits can lead to pitting corrosion [“localized corrosion resulting in pits, i.e. cavities extending from the surface into the metal” (ISO 8044) (Table 1)]. These pits, or the complex geometries of the deposited metals that form, can create areas shielded from the bulk fluid/electrolyte. Subsequent hydrolysis of metal ions creates an acidic medium and attracts charge-neutralizing ions such as chloride and sulfate, resulting in self-sustaining pitting that, if it occurs in crevices, is called **crevice corrosion** (Table 1).

**Table 1.** Brief description of the main mechanisms associated with MIC of metals.

MIC mechanisms	Description	Diagram
**Under deposit corrosion, oxygen gradient corrosion**	A type of “localized corrosion associated with, and taking place under, or immediately around, a deposit of corrosion products or other substance” (ISO 2020), e.g. biofilm or metal deposition by metal-oxidizing bacteria that is formed in a patchy arrangement.	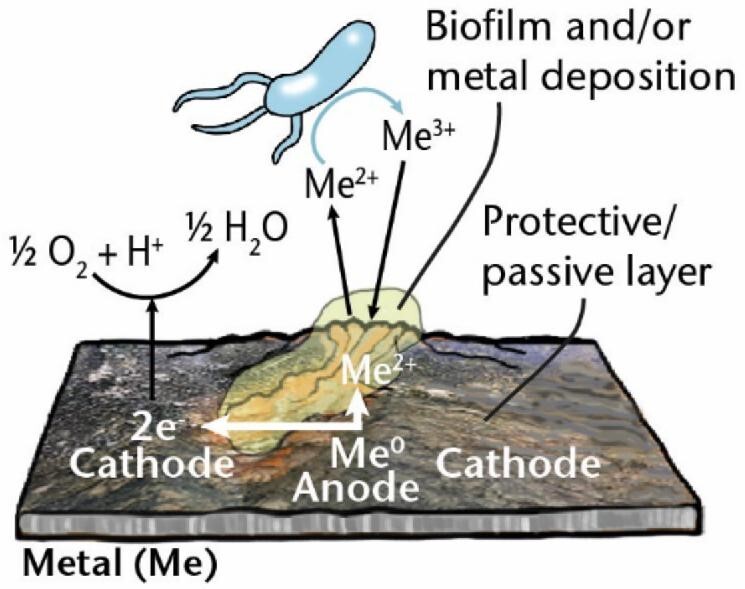
**Crevice corrosion**	A type of “localized corrosion associated with, and taking place in, or immediately around, a narrow aperture or clearance formed between the metal surface and another surface (metallic or non-metallic).” (ISO 8044) The accumulation of chloride and other aggressive anions in the pit accelerates corrosion.	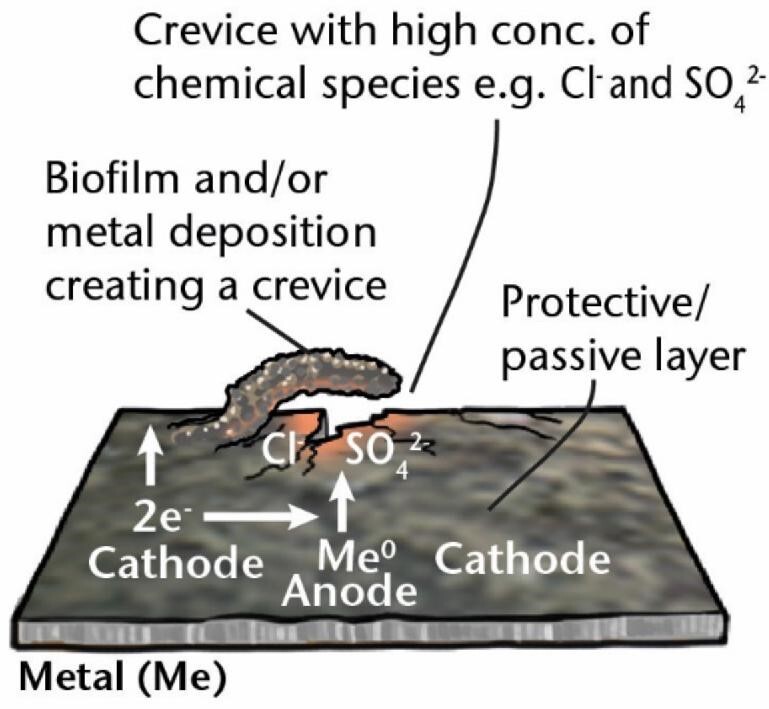
**Electrical MIC (EMIC)**	Corrosion caused by extracellular electron transfer by microorganisms.	
***Direct EMIC***	Corrosion of metals achieved by extracellular electron transfer by microorganisms in direct contact with the metal surface. Electrons are taken up by cell surface enzymes or membrane redox proteins.	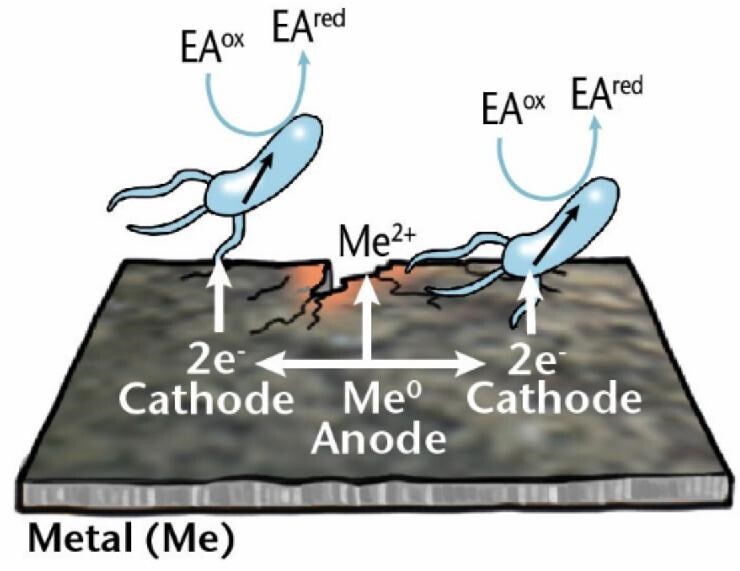
***Indirect EMIC***	Corrosion of metals accelerated by soluble electron transfer mediators released from microorganisms that use the electrons gained from the metal for respiration.	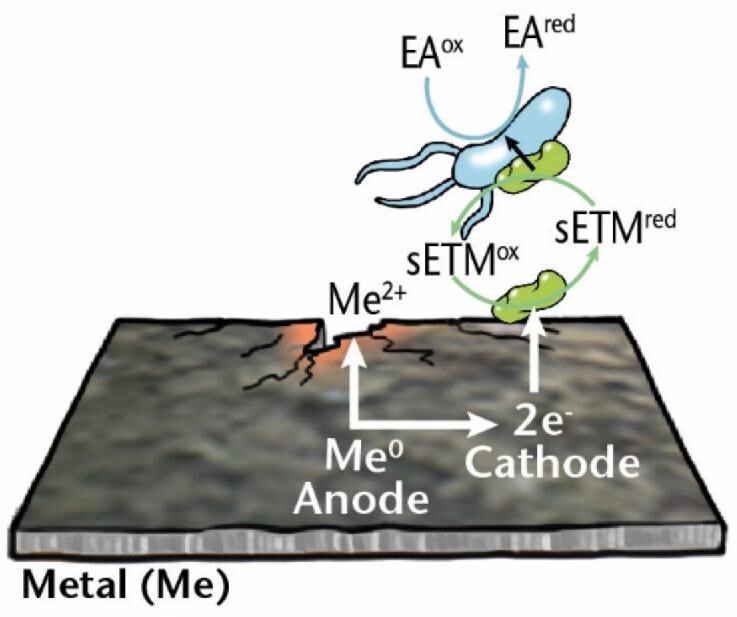
**Metabolite MIC (MMIC)**	Corrosion of metal achieved directly or indirectly by metabolites released by microorganisms both in aerobic and anaerobic conditions.	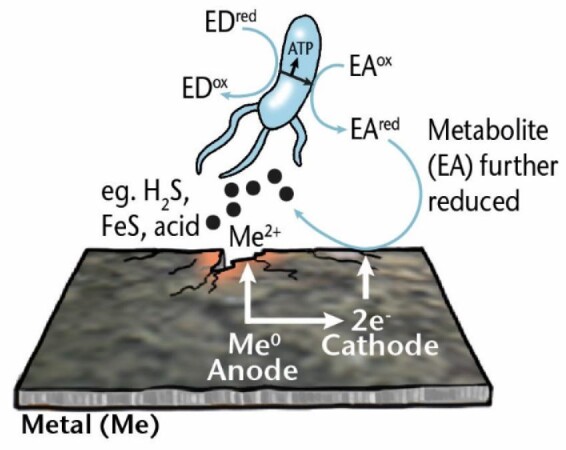

*Me: metal; EA: electron acceptor; ED: electron donor; ox: oxidized; red: reduced; sETM: soluble electron transfer mediator; MeOB: metal oxidizing bacteria.-Test*

Some materials, such as corrosion-resistant steels, have enhanced resistance to oxygen corrosion as their passivated surface (thin metal-oxide layer; see the Materials and MIC section below) provides protection. However, some biofilms can destroy this protective layer, resulting in pitting corrosion (Yuan and Pehkonen 2007, Li et al. 2016, Dong et al. 2018, Cui et al. 2022). In addition to the formation of a biofilm, the respiration of aerobic bacteria within a biofilm can reduce the oxygen content, creating an anaerobic environment that can support the growth of anaerobes such as sulfate-reducing prokaryotes (SRP) and nitrate-reducing prokaryotes (NRP).

### Electrical MIC (EMIC)

EPS secreted by microbial cells have many components with redox properties and electrochemical (EC) activity that play crucial roles in microbial respiration as well as in corrosion. For example, sessile cells in a biofilm can use metal, such as elemental iron, as an electron donor if thermodynamically more favorable electron donors are lacking (Philips et al. 2018). In anoxic environments, the terminal electron acceptor is an oxidizing agent such as sulfate or nitrate. While the reduction of the electron acceptor takes place inside the cell, the oxidation of the electron donor happens outside the cell, so the extracellular electron from outside must enter the cell. This electron transport across the cell wall is called *extracellular electron transfer* (EET), and the overall mechanism through which the associated corrosion of metal is achieved is called *EMIC* (Enning and Garrelfs 2014), alternative names include *type I MIC* (Gu 2012) and EET-MIC (EET MIC) (Jia et al. 2019). If the electron donor is an organic carbon source that can diffuse into the cell, there is no need for an electron transport mechanism because the electrons released are already located in the cytoplasm. Insoluble metals, such as elemental iron, cannot pass through the cell membrane, and an extracellular electron transport is required for it to be used as an electron source. The electron can be transported into the cell in two ways, namely by *direct* or *indirect* mechanisms (Table 1).

In *direct EMIC, direct extracellular electron transfer* (DEET) (Lovley 2011) occurs, the cell can have direct contact with the metal surface and directly accept electrons from the metal. Until recently, this mechanism had only been inferred, but Tang et al. (2019) provided evidence for iron as a direct electron donor. Electrons are taken up by cell surface enzymes, structures, or membrane redox proteins, such as c-type cytochromes (Paquete et al. 2022), facilitating EMIC. Alternatively, the cell can attach to the metal by “nanowires,” e.g. electrically conductive pili in bacteria (Lovley 2017) or archaella in archaea (Walker et al. 2019), by which it can transfer electrons. The exact mechanism for electron transfer through electrically conductive cell appendages is still debated, and Little et al. (2020a) argue that any electronic transport via pili is unlikely to significantly contribute to MIC. Thus, further research is needed to resolve the role of electrically conductive pili in MIC.

From an EC standpoint, the term “direct electron transfer” is possibly not strictly correct as each mechanism described above requires a redox mediator. Blackwood (2018) has contested that true direct electron transport does not and cannot happen, as direct electron transfer between aqueous species cannot occur over distances of >2 nm. This is a typical example of different disciplines using different language to describe a phenomenon, increasing the chance of misunderstanding and confusion. This confusion is even further increased by alternative terms and their abbreviations for direct EMIC, including DET-MIC (direct electron transport MIC) (Lekbach et al. 2021) or DIMET (direct iron-to-microorganism electron transfer), if direct electron transport occurs from Fe^0^ (Lekbach et al. 2021), along with the other alternative terms for EMIC as indicated above. Despite the controversies and mixed terminologies on the different categories of direct MIC, we use the umbrella term “direct EMIC” here when referring to microorganisms capable of directly interacting with the metal surface by one of the various proposed mechanisms.

In *indirect EMIC*, soluble electron transfer mediators (Huang et al. 2018, Tsurumaru et al. 2018) are released from the cell, oxidized at the anode, and return back to the cell to be used in respiration (Kato 2016). Alternative abbreviations also exist for this mechanism [*MEET* (mediated EET (Little et al. 2020a); *MET-MIC* (mediated electron transport MIC) (Gu 2012); and *SIMET* (shuttle-mediated iron-to-microorganism electron transfer), if iron is the electron source (Lekbach et al. 2021)].

### Metabolite MIC (MMIC)

In metabolite-MIC, microorganisms influence corrosion through the creation of corrosive metabolites, such as protons, organic acids, or sulfur species. These metabolites are reduced on the metal surface, and a biocatalyst is not required for the process, as opposed to EMIC. At a sufficiently low pH, proton reduction can be coupled with metal oxidation. This type of corrosion mechanism is also an EC process. Alternative terms for MMIC include *type II MIC* (Gu 2012) and *chemical MIC* (CMIC) (Enning and Garrelfs 2014). Li et al. (2018) argued that the term “metabolite-MIC” is preferable over “chemical MIC,” because chemical corrosion is the direct reaction of a metal with an oxidant, usually at high temperatures, with no separable oxidation and reduction reactions, as opposed to EC corrosion.

### The historical cathodic depolarization theory

Historically, many MIC mechanistic studies in the absence of oxygen were reported in relation to sulfate-reducing bacteria (SRB), and the associated severe corrosion was often explained by the cathodic depolarization (CDP) theory first proposed by von Wolzogen Kühr and Van der Vlugt (1934) (translated into English in 1964). According to the CDP theory, the rate of iron corrosion by SRB is increased by the removal of H_2_ from the cathode by hydrogenase-containing SRB. To be precise, in the absence of oxygen, the electron acceptors for iron oxidation are protons derived from dissociated water, whereas in the cathodic reaction, the proton is reduced to H_2_. According to the theory, the H_2_ formed on the metal surface is consumed by SRB and thereby further accelerating iron oxidation.

The CDP theory has been criticized for decades and been discredited by many (Hardy 1983, Crolet 1992, Dinh et al. 2004, Mori et al. 2010), and reviewed in detail (Enning and Garrelfs 2014, Blackwood 2018). In short, the rate-limiting cathodic reaction in metal corrosion is the adsorption of protons to the metal, not the desorption (removal or dissolution) of H_2_ from the surface, i.e. in abiotic cultures, low corrosion rates are due to the limited availability of protons and, thus, slow H_2_ formation on iron. It has been shown that the consumption of cathodic hydrogen by SRB did not significantly increase iron corrosion in the presence of iron as the sole electron donor (Venzlaff et al. 2013). This does not rule out the possibility that the utilization of hydrogen by microorganisms may still play a role in MIC, but not as it was proposed/intended by the CDP theory. Overall, it is recommended that in the future, the CDP theory should only be mentioned if needed for historical purposes, and if it is mentioned, the controversy and criticisms of this explanation for MIC should be acknowledged.

To summarize, MIC is not a single corrosion mechanism. Instead, several different mechanisms can contribute to MIC. However, the two main common features that are similar in all MIC cases are that (1) microorganisms play a role and (2) MIC is an EC process.

## Siloed scientific fields and the need for interdisciplinary dialogue

While MIC by definition encompasses the fields of microbiology and corrosion, the involvement of other disciplines such as electrochemistry, production chemistry, metallurgy and materials science, process engineering, fluid mechanics, and others is essential to getting a clear picture of the environments and operating conditions that support MIC. As early as 1934, while leading a group of scientists in the field study of a sphagnum bog, Baas Becking (Baas Becking and Nicolai 1934) observed that simply classifying the microorganisms present would yield “less satisfaction to the investigator” than it could have with additional scientific insights from geologists, geneticists, and ecologists. Decades later, a review of MIC state-of-the-art in 2005 by Videla and Herrera (2005) noted that until the late 1970s there was poor transfer of knowledge between disciplines, including metallurgy, electrochemistry, microbiology, and chemical engineering, that prevented the study of MIC from going much beyond a focus on SRB/SRP. Today, it is becoming more widely understood that any investigation of MIC requires a multidisciplinary focus on multiple lines of evidence (MLOE), as reflected in Sharma et al. (2022), where data from molecular methods were analyzed and conclusions drawn by a multidisciplinary team. Yet, when industries today are attempting to understand the impact of MIC on their assets, many do not have experts from multiple disciplines on hand to guide their sampling, testing, and data integration to help them solve complex MIC issues. Further, while there are numerous standards available to guide specific types of testing, there are none that identify a truly unified multidisciplinary approach for combining MLOE to ultimately direct MIC management activities.

### Diagnosing MIC requires MLOE

The diagnosis of MIC requires MLOE for a number of reasons, but perhaps foremost is the fact that there exists no singular test or assay that can conclusively identify that MIC has occurred or is presently occurring, although some current works, e.g. Lahme et al. (2021), have suggested that [NiFe] hydrogenases in methanogenic archaea can be potential MIC biomarkers under specific conditions. Early MIC work in the oil and gas industry was heavily focused on the use of culturing methods (such as the most probable number—MPN—technique) and relating the likelihood of MIC to culturable cell counts in various types of media. Gradually, and with the increased application of molecular microbiological methods (MMM), asset owners discovered that while microorganisms were present nearly everywhere, the cell counts poorly correlated with actual MIC damage (Zintel et al. 2003).

**Figure 1.**
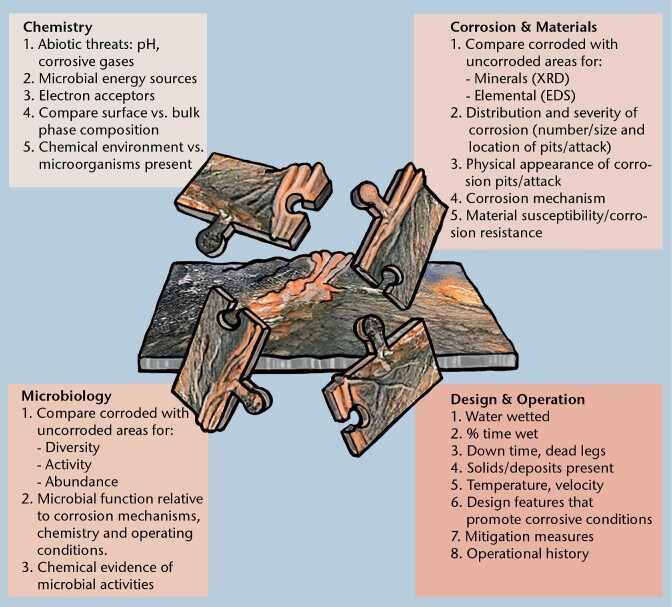
MLOE used in the MIC assessment. Puzzle pieces represent the four main categories of evidence with typical types of measurement. To solve the puzzle, evidence from most or all four categories is needed.

Another reason why MLOE are used for MIC diagnosis is the lack of a unifying model or equation to calculate corrosion rates due to MIC, which is made only more difficult by the fact that, and as explained in the previous section, microorganisms and biofilms can affect corrosion reactions in a variety of ways. In addition, MIC can at times be linked with corrosion caused by abiotic factors. The MLOE approach is common to many other scientific fields, including but not limited to the study of sediments, microbial fuel cells, and bioremediation. The approach is a “systems” view of microbial ecology, including the roles of the chemical environment and the role of the material being degraded or deteriorated. Figure 1 shows an example of the four MLOE categories often used for MIC investigations, including examples of parameters that are included in each category. The more pieces of the puzzle that can be provided, the better the picture/understanding of what is happening will be.

The best diagnosis of MIC requires MLOE from as many of the four categories shown in the puzzle as possible. Evidence from more categories provides increased confidence. At present, this is still a work in progress, and there are no definitive guidelines on which tests or combination of tests provide the best evidence. According to Lee and Little (2017), the goal is to collect independent types of measurements that are consistent with a MIC mechanism.

To obtain MLOE, the investigation of corrosion in an asset would ideally be based upon proper characterization of (1) the conditions that are present in the “bulk” environment (e.g. soil, water, process fluid); (2) any biofilm or other material at the metal/environment interface and associated physicochemical conditions, most likely to be involved in MIC; and the (3) the metal surface itself, both where corrosion has formed and where it has not. These three environments can be quite different from one another, even though they are present at the same time in the same place. Wrangham and Summer (2013) showed that the types and numbers of microorganisms present in a bulk fluid phase can be quite different than those located within a biofilm on a surface. Deposits on a metal surface can also vary in composition and physical properties throughout their thickness and laterally, as demonstrated by Larsen et al. (2010), who showed significant differences in both corrosion product composition and microbiology in thick deposits inside of pipework on an offshore oil and gas production platform.

Obtaining as much information as possible from a combination of the different environments present is important to get the most accurate understanding of the overall processes taking place. If only limited testing is available, it is recommended the focus of testing should be on the metal interface, as this is where the key corrosion interactions are likely taking place.

### The roles of engineering design and operations in MIC assessment

It is valuable for microbiologists to have an understanding of the overall operation for assets where MIC is being assessed. Engineers, operators, maintenance personnel, production chemists, and chemical vendors can provide valuable insights that reveal when and why environmental changes occur. A simple example is the operating temperature. Operations may report that the crude oil production temperature is 80°C, leading microbiologists to look for thermophiles as a possible cause of MIC; however, it would also be important to know that the process only runs once a month for a day, then cools down to ambient temperature. Any factor in the design, operation, or maintenance of an asset that can affect the chemical and microbiological environment should be an area of interest, and microbiologists may need to prompt other experts to obtain this type of information; it may not be volunteered otherwise. It is particularly important to understand the types and doses of various treatment chemicals that may be used, and production chemists and corrosion engineers can generally provide this information. Changes to asset design, the fluids being received or processed, the corrosion mitigation measures being applied, increases in newly found corrosion, etc. can all provide important insights to microbiologists working to solve a MIC issue. Leak and failure histories, particularly if root cause analysis has been performed, can also provide useful context when assessing MIC (Borenstein and Lindsay 2002, Eckert and Skovhus 2018, Gősi et al. 2022).

Engineering and design information is also valuable when assessing the potential for MIC and abiotic corrosion mechanisms. Such information includes the type and grade of materials used for construction, fabrication, and testing history; circuits and systems identified on engineering drawings, process flow diagrams, and mass balance sheets; identification of dead legs (where flow infrequently occurs); clean-out capabilities for large vessels; flow controls; and utilities supporting various processes in the assets. Often, MIC is found in piping and assets with no flow or stratified flow, which allows solids and water to accumulate and promote the growth of biofilms (Sharma et al. 2022). A review of operation and design parameters can help to identify such areas to guide inspection and mitigation activities. Additionally, older assets may no longer conform to the operating conditions used as the basis for design, which affects the type and severity of likely corrosion threats (Wei et al. 2022). Table 2 provides some examples of operational and engineering information that can help support MIC threat assessments.

**Table 2.** Operational and engineering information that can help support MIC threat assessment.

Engineering/Design Information	Operational Information
Material dimensions and wall thickness. Corrosion allowance	Year of installation / commissioning
Material grade and manufacturer; suitability of the material for process fluids and conditions	Operational problems – solids accumulation, flow restriction, unplanned outages
Methods of fabrication, joining e.g., welding procedures, weld quality assurance and inspection, heat treatment; use of flanged connections (crevice formation)	Fluid characteristics and chemical composition; including sample locations and means of sample collection and preservation, field and lab analytical methods and procedures used
Design code; engineering drawings, process flow diagram	Field modifications to original design; management of change records
Design operating window (temperature, pressure, flow rate)	Actual operating window (temperature, pressure, flow rate)
Pipeline elevation profile Results of any flow modelling that was performed Inputs and outputs; systems and circuits	Actual process inputs and outputs Microbiological monitoring data, including sample locations, sampling and preservation methods, field and lab methods and procedures used
Corrosion circuits and initial corrosion threat assessment for design	Corrosion monitoring results from coupons and probes
Identification of dead legs, no-flow areas	Leak/failure and repair history
List of intended corrosion monitoring locations	Corrosion mitigation – actual activities, chemical treatment, pigging, flushing, etc.
Initially identified corrosion mitigation measures to be applied	Inspection and maintenance records, integrity assessment records
Means of pre-commissioning testing; hydrostatic test records, procedures, actual test media used	Process upsets, emergency shut down records-Test

### Chemistry: assessing the chemical environment

Non-microbiologists generally do not have the same perspective on the chemical environment as microbiologists, e.g. the significance of different electron acceptors, energy sources, pH, redox potential, salinity, and other factors that affect microbial ecology (Skovhus et al. 2017). The concepts of exponential growth, the widespread diversity of microbiomes, the essential inputs to (and end products from) microbial metabolism, and the important roles of biofilms and EPS are somewhat foreign to experts in other disciplines (Wade et al. 2023). Microbiologists may have the opportunity to help other disciplines to view information related to MIC through the “lens” of microbial ecology. Likewise, a dialog with chemists, corrosion engineers, and operators can bring new insights to those focused on the microbiological environment (Hashemi et al. 2017). It is imperative that all parties in the multidisciplinary conversation have a clear understanding of the technical terms that are being used, as each discipline typically has its own technical vocabulary (Eckert and Skovhus 2018).

The chemical composition as well as the physical parameters of the environment in which MIC or abiotic corrosion occurs are very significant, in that the chemistry of the bulk phase environment and of surface films/deposits impacts both electrochemistry and microbiological processes. While sampling and chemical/physical analysis of the bulk phase (e.g. aqueous) is fairly straightforward, analysis of chemical conditions at the metal surface, particularly beneath solid particles and biofilms is considerably more complex (Phull and Abdullahi 2017, Kromer et al. 2022). When analyzing chemical composition data, it is imperative to keep this distinction in mind; e.g. the pH in the bulk phase may be considerably different from the pH beneath a biofilm containing acid-producing microorganisms (Lee et al. 1993, Dexter and Chandrasekaran 2000, Phull and Abdullahi 2017). There are many commonly used analytical methods for water composition, dissolved gas, and headspace gas analysis, and some examples of these are detailed here.

### pH

pH is an essential measurement parameter in the aqueous phase of a system affected by MIC (Ibrahim et al. 2018). Changes in the pH may indicate the growth of acid-producing microorganisms, such as acetogens, or partial pressure variations, such as dissolved O_2_ and CO_2_ concentrations (Lee et al. 1993, Mand et al. 2014, Kato 2016). In a given field environment, the local pH condition has a direct impact on the microbial community and activity; e.g. the corrosive acetogen *Sporomusa sphaeroides* thrives in pH ranging between 6.4 and 7.6 and can tolerate up to 8.7 (Philips et al. 2019), whereas some SRB can grow up to pH 9.5 but with an optimal pH range around 7 (Ibrahim et al. 2018). One key challenge is understanding the actual pH in a given environment, as pH is affected by temperature and pressure (Phull and Abdullahi 2017). For example, a sudden shift in system pressure, such as liquid withdrawal from a pressurized system, will alter the pH measured (Ibrahim et al. 2018). According to the Henry’s Law, gas solubility is directly proportional to the partial pressure, which is particularly important for CO_2_ and H_2_S. Changes in their solubility will also influence the pH of the aqueous environment, that subsequently impact microbial growth and corrosion product formation (Ibrahim et al. 2018). Metal dissolution and corrosion product formation are intertwined with biofilm and influenced next to others by pH. For example, the formation of FeCO_3_ (siderite) is more stable in higher pH, since the concentrations of HCO_3_^−^ and CO_3_^2−^ are higher than the respective iron ions, thus favoring the crystallization of siderite (Joshi 2016). Furthermore, pH can act as an indicator for MIC mitigation. The topic of MIC mitigation is discussed below in the Corrosion Management section.

### Concentration of dissolved ions

Concentrations of cations and anions in the aqueous phase can also indicate possible microbial activity. For example, depletions in the concentrations of electron acceptors such as nitrate and sulfate indicate the activities of NRP and SRP, respectively. The mass balance between the cations and anions of a given environment provides useful evidence for evaluating the overall MIC process, including the metabolic process, corrosion product deposition, and metal dissolution. For example, the concentration of sulfur species in the aqueous phase, including S_2_O_3_^2-^, SO_4_^2−^, HS^−^, SO_3_^2−^, and S^0^, is closely related to the oxygen concentration in a system and microorganisms such as sulfur-oxidizing bacteria and SRB (Ibrahim et al. 2018). Correct dosages of biocide and nitrate injection to combat MIC also require close monitoring of cations/anions (Gieg et al. 2011, Ibrahim et al. 2018). For example, in the oil and gas industry, for nitrate injection to be successful, the concentration of NO_2_^−^ needs to remain stable in the system to inhibit the activities of SRB, as the further reduced compounds of NO_2_^−^ in the metabolic pathway of NRB, namely N_2_ and ammonia, are ineffective against SRB. Thus, a close monitoring of the anions NO_3_^−^, NO_2_^−^, HS^−^, and SO_4_^2−^ will provide a detailed overview on the efficacy of nitrate injection on the activities of SRB. One key challenge is the timely measurement of the associated ions; e.g. HS^−^ ions are highly volatile and can quickly escape into the atmosphere post-sampling (Tangerman 2009). It is important to ensure on-site readily available measurements when conducting analyses of key ions. In addition, the differences in the levels of cations and anions provide important evidence for the corrosion product formation process. For example, a decrease in the level of carbonate ion and Fe^2+^ in the aqueous solution may indicate the formation of FeCO_3_, and the respective concentrations of the ions are used for calculating the supersaturation index (SS) (Joshi 2016):


(1) \begin{eqnarray*}
S{S}_{FeCO3} = \frac{{\left[ {F{e}^{2 + }} \right]\left[ {CO_3^{2 - }} \right]}}{{{K}_{sp}\left( {FeCO3} \right)}}
\end{eqnarray*}

where the K_sp_ is the solubility product constant for FeCO_3_ and an SS value of above 1 indicates that the solution is saturated (Joshi 2016). Overall, the mass balance between the various cations and anion species is a strong indicator of the MIC process.

### Gas production

Several known microorganisms associated with MIC produce biogenic gas. For example, the corrosive methanogens produce methane using the electrons from the metal surface (Beese-Vasbender et al. 2015, An et al. 2020, Tamisier et al. 2022), whereas SRB activities lead to the production of H_2_S. Gas chromatographs (GC) equipped with a thermal conductivity detector or flame ionization detector are typically used for gas analyses (Grob and Kaiser 1982). In the field of MIC, it is noteworthy that multiple biogenic gases may need to be monitored, including CO_2_, CH_4_, H_2_S, H_2_, O_2_, N_2_, etc. Hydrogen is one of the key gases of special importance to MIC, as several corrosive species are dependent on H_2_ for their growth. In addition to GC, various handheld and in-line H_2_ sensors are commercially available that allow field monitoring of H_2_ and are particularly useful during field sampling (Boshagh and Rostami 2020). However, such devices can be limited in resolution, and their reliability can be affected due to contamination by other gases or wrong handling in the field. For extremely local environments, such as within the metal-biofilm interface, monitoring of the H_2_ gradient can be performed using microsensors (Cai et al. 2020b). While the current H_2_ microsensor technologies are still evolving to reduce interference from H_2_S and other compounds (Nielsen et al. 2015), local monitoring of H_2_ remains an important line of evidence during MIC investigations and monitoring.

### Microbiology: assessing microbiological composition and activity

The microorganisms associated with corrosion are, of course, strongly linked to the chemical and physical environmental conditions present, but microbiological activities also affect the local environment in terms of organic or mineral acid production, sulfide production, or the formation of occluded areas and concentration cells on the metal surface. Little et al. (1996) demonstrated that microorganisms in biofilms can do both: create local anodic areas and are also “attracted” to existing anodic sites previously unaffected by microorganisms. Non-microbiologists can easily become lost in the complexity of interactions that could be occurring between various microorganisms in biofilms, their metabolic capabilities, and the kinetics that are driving reactions in one direction or the other. This is compounded by a lack of comprehension of the strengths and limitations of different microbiological characterization methods/technologies, the issues of interference, primer coverage, biases, sensitivity, etc. It is often stated that microbiological conditions may be described in terms of diversity, enumeration, and activity. Engineers are generally not aware of the difficulty in determining the specific microbial activities that are occurring in a given environment, e.g. using RT-qPCR or metabolomics, and that these activities are dynamic, changing in parallel with environmental conditions. Microbiologists, with expertise on these and other associated topics, can provide essential insights on such matters to non-microbiologists.

Over the years, various techniques have evolved and been gradually replaced by more advanced technologies to investigate MIC, all the way down to the molecular level (as reviewed in Little et al. 2006, Beale et al. 2016, Trif et al. 2018, Kotu et al. 2019). In support of this observation, Puentes-Cala et al. (2022) overviewed the MIC literature published in the last 12 years, which showed that approximately three-quarters of the studies used molecular microbiological approaches to characterize microbial communities in field samples. Table 3 summarizes some of the traditional as well as more advanced methods that can be used to obtain microbiological data, highlighting their pros and cons to aid decision-making during MIC investigations. All of these techniques are suitable to use for both field and laboratory studies if handling is done properly, as described elsewhere [e.g. in Eckert (2022) et al. (2022) or AMPP Standard TM21465 (under preparation)].

**Table 3.** Example of methods used to obtain microbiological data for MIC studies.

Method	Pros	Cons	References
**Microbiology**			
Culture based methods			
*Plate counts*	Low cost. By using selective medium, types of microorganisms can be counted and isolated.	<1% of microorganisms can be cultured; very limited information on taxonomy. Takes several days to get results. Molecular approaches are needed to confirm results.	(Health Canada 2013)
*Most Probable Number (MPN)*	Low cost. Easy to use.	Fails to recognize activity. Many MIC related microorganisms are non-culturable, cannot be detected by MPN.	(Jensen et al. 2013; Skovhus et al. 2017)
*Test kits to enumerate MIC related microorganisms*	Easy to use. No trained personnel are needed.	Low specificity. Takes days to get results. False results occur. Use of circumstances has to be carefully determined.	(Phan et al. 2022)
**Microscopy**			
*Light microscopy*	Easy to use. Low cost.	Mere detection of microorganisms is not diagnostic for MIC.	
*Epifluorescence Microscopy*	Useful for testing effectiveness of biocide killing or to show the presence of microorganisms at the site of corrosion.	Sample needs to be fixed and stained. Often difficult to differentiate cells from background material.	(Dang et al. 2022)
*Confocal Laser Scanning Microscopy (CLSM)*	By using specific staining, the biofilm on the surface can be visualized.	Skilled personnel are needed.	(Arun et al. 2020)
*2D and 3D Optical Coherence Tomography (OCT)*	No staining required. Relies on specific imaging techniques to assess the structural properties of biofilms.	Skilled personnel are needed.	(Lima et al. 2022; Romeu et al. 2022)
*Electron Microscopy (EM)*	Visualization of microorganisms/biofilm at the site of corrosion. Direct contact of microorganism with metal can be shown. If coupled to EDS (Energy dispersive X-ray analysis), elemental composition of corrosion products can be defined	Skilled personnel are needed. During SEM analysis, the original structure of biofilm is disturbed.	(Ray et al. 1997)
*Atomic Force Microscopy (AFM)*	Higher resolution than SEM.	Relatively small scan areas, skilled personnel are needed.	(Xu et al. 2002)
**DNA/RNA/ protein based techniques**			
*Hybridization methods, e.g. FISH*, DNA microarrays	Quantitative. Selected genes (groups of microorganisms e.g. SRB, SRA, Bacteria, Archaea) can be visualized.	Prior knowledge of the microorganisms to be detected is needed. Trained personnel are needed.	(Kotu et al. 2019; Skovhus et al. 2009)
*Polymerase chain reaction (PCR) / quantitative PCR (qPCR)*	Cheap and fast alternative. Can provide results in a few hours.	Genes have to be selected for amplification, unknown species remain unidentified. Skilled personnel are needed.	(Lahme et al. 2021; Parow et al. 2022)
*Amplicon sequencing*	Specific for bacteria or fungi at genus or species level. Less costly than shot-gun metagenomics.	Contamination is amplified (amplification bias). Less taxonomic resolution. Functional profiling is not possible.	(Sharma et al. 2022)
*Shot-gun metagenomics*	High specificity. High taxonomy resolution. Functional profiling is possible. Covers viruses, bacteria, archaea and eukarya at species or strain level.	Does not differentiate between active or passive cells. Can be costly, though getting cheaper. Samples have to be sent to specialized laboratories for analysis.	(Bonifay et al. 2017; Gősi et al. 2022)
*Metatranscriptomic*s	Provides valuable information about gene activity (expression).	Larger sample size is needed to perform analyses as it is more difficult to isolate RNA than DNA. Samples are more sensitive to degradation. Can be costly and a specialized laboratory is needed. Skilled personnel are needed for data interpretation.	(Krohn et al. 2021)
*Metaproteomics*	Allows the identification of proteins produced by microbial strains or communities.	High number of unassigned peptides, lack of well-annotated protein databases. Requires trained personnel and costly equipment. Large cell number is needed for analysis.	(Chatterjee et al. 2021; Dupree et al. 2020)
*Metabolomics*	Allows identification and quantification of metabolites related to MIC.	Can be costly and specialized laboratory is needed. More than one analytical method may be needed to identify all metabolites present. Special care is needed in sample handling. MIC-specific metabolites have not yet been clearly identified.	(Bonifay et al. 2017; Mand et al. 2016)
**Enzymatic methods**			
*ATP assay method*	Easy to use, fast measure to provide estimation of microbial inhibition, metabolic state of microorganism and total biomass.	Does not provide information about the composition of the biomass.	(Dockens et al. 2017)-Test

It is imperative to emphasize that the limitations of each microbiological method should always be considered, as, e.g. the detection of microorganisms that have been associated with corrosion by itself is not diagnostic for MIC (Little et al. 2006). Also, the choice of methods should carefully be evaluated in light of the asset affected, the main questions to be answered, the availability of trained personnel to perform the sampling, and/or access to relevant laboratories. The methods presented above could be used individually or in various combinations to provide the best possible line of evidence to assess the involvement of microorganisms in corrosion. Furthermore, these methods, either used alone or in combination, are not sufficient to support the involvement of microorganisms in a corrosion process; collecting other lines of evidence, e.g. chemical, metallurgical, and operational information, is critical during MIC diagnosis/studies.

### Materials and MIC

There are a few high-level points for microbiologists to consider when thinking about corrosion mechanisms and electrochemistry. The first is that abiotic or non-biological corrosion reactions need to be considered in every MIC evaluation. Abiotic corrosion may be present separately from, or in conjunction with, MIC. Microorganisms, e.g. could be forming biofilms that simply create more crevices for differential aeration corrosion cells, leading to localized pitting on passive materials such as stainless steel (SS) (Table 4). The second consideration is that microbial activity in a biofilm may simply enhance the effects of existing and well-known abiotic metallurgical conditions that can promote localized corrosion, such as the effects of manganese sulfide inclusions forming microscopic anodic corrosion initiation sites or galvanic corrosion occurring where metals having differing native potentials are joined (e.g. carbon to SS). It is important for microbiologists to understand these abiotic contributors to corrosion when examining the role of microorganisms in the corrosion of a given material, and metallurgists and materials scientists can readily explain these contributors. Finally, and probably one of the more elusive challenges, is developing an understanding of how microbiological metabolism facilitates or enhances the kinetics of anodic and cathodic corrosion reactions that must be occurring for corrosion to take place. As one electrochemist recently stated in an MIC symposium, “I need to know where the electrons are going!”. This is an area where there is significant room for improvement in our understanding of MIC; however, one that will require a serious collaborative effort between materials scientists and microbiologists to make significant progress.

**Table 4.** Examples of engineering materials and reports of their susceptibility to MIC [Note: where possible, references showing examples of MIC of materials in the field or reviews have been included].

Metal	Examples applications	MIC examples
Iron-based alloys		
Carbon steel	General infrastructure, piping, tanks, aircraft and shipping structures	(Croese et al. 2018; Gősi et al. 2022; Jacobson 2007; Li et al. 2001; Mara and Williams 1972; Usher et al. 2014)
Cast iron	Piping, machine parts	(Kajiyama and Koyama 1997; Smart et al. 2014; von Wolzogen Kühr and Van der Vlugt 1934)
Galvanized steel	General infrastructure, sprinkler systems	(Bolton et al. 2010; Ilhan-Sungur et al. 2015)
Marine-grade steel	Ship hulls, harbor walls, wharf structures	(Huang et al. 1997; Melchers and Jeffrey 2013; Melchers et al. 2014)
Stainless steel	Piping, tanks, aircraft and automobile structures, general infrastructure, medical devices	(Borenstein 1994; Huttunen-Saarivirta et al. 2012; Ibars et al. 1992; Javed et al. 2020; Liu 2014; Moreno et al. 1994; Trampus et al. 2017);
Copper-based alloys	Piping, heat exchangers, wiring, general infrastructure, ship hulls, statues	(Amendola and Acharjee 2022; Dou et al. 2018; Javed et al. 2016a; King et al. 2021; Little et al. 1990)
Aluminum	General infrastructure, tanks, aircraft and automobile structures, piping, ship hulls	(Dai et al. 2016; Hagenauer et al. 1994; He et al. 2022; Smirnov et al. 2008; Starosvetsky et al. 2007)
Nickel-based alloys	Marine and engine components	(Gouda et al. 1993; Little et al. 1990; Wade et al. 2018)
Magnesium	Aircraft and automobile components, sacrificial anodes	(Lan et al. 2022; Qu et al. 2015; Zhu et al. 2014)
Titanium	Aircraft structures, piping, medical devices	(Costa et al. 2021; Little et al. 1993; Rao et al. 2005)-Test

In addition to the chemical, microbiological, and physical environment, the potential for MIC depends on the composition and metallurgical properties of the material being affected by these parameters. Carbon steel (CS) and concrete are two of the most predominant materials used in the construction of engineered assets, including pipelines, sewer and water lines, marine structures, ships, offshore energy generation, and infrastructure, such as bridges and highways. Concrete and the CS reinforcing used within the concrete are often subject to corrosion, although the percentage of this corrosion resulting from MIC other than in sewer lines (Wu et al. 2020) is not well understood. There is, however, a long history of research and information published about the interaction of metals with biofilms. The aim of this section is to provide a general introduction to materials and, specifically, metal properties that are relevant to MIC.

Metals can generally be broken down into two categories, i.e. passive and active metals, depending on the metal and the environment to which it is exposed. Passive metals, e.g. corrosion-resistant alloys (CRA) like SS, form a protective metal oxide when exposed to aqueous environments containing oxygen. Active metals, such as CS, do not form this protective layer when exposed to aerated water. Typically, passive metals perform better in relation to corrosion; however, this is not always the case. Many metals used in industrial applications are alloys (a combination of elements), where small changes of compositions can make significant performance differences. The processes used for manufacturing metals (e.g. temperatures, mechanical processing) can also affect the microstructure of essentially the same alloy, which can affect corrosion. In addition, construction and fabrication processes such as welding can also adversely change the properties of metals to make them more susceptible to corrosion. Each of these factors can also affect the likelihood and magnitude of MIC that may occur.

Table 4 shows some examples of alloy categories and typical applications (Pierre R. Roberge 2012), along with references where MIC case studies of these materials can be found.

Since MIC most often results in localized corrosion (pitting), a metal’s resistance to pitting is important to engineers and designers seeking to prevent MIC. One indicator of pitting resistance in SSs is the pitting resistance equivalent number (PREN), which is based on a calculation using the amount of chromium, molybdenum, and nitrogen present in an alloy. PREN is used to compare the relative resistance of alloys to pitting corrosion in chloride-containing aqueous environments. Alloys with a PREN of 32 or greater are generally considered to be resistant to pitting corrosion in ambient-temperature seawater. A material’s PREN value may also provide some level of insight in determining its relative resistance to MIC (Eckert and Amend 2017 et al. 2017); however, care needs to be taken not to over-interpret this value (Craig 2020). A general review of the literature in which MIC is cited as the cause of corrosion will show that as PREN increases, the frequency of MIC case studies decreases. MIC is frequently reported for CSs and somewhat less frequently for SS. For duplex (DSS) and super-DSS SSs, nickel-based and titanium alloys the incidence of reported MIC is fairly rare.

In laboratory studies using *Desulfovibrio desulfuricans*, 2205 DSS was reported (Antony et al. 2007) to experience etching, pitting, and crevice attack after 40 days exposure in a chloride-containing medium. Another study (Machuca et al. 2012) of DSS in natural sea water showed crevice corrosion only occurred in samples that were electrochemically polarized (Machuca et al. 2012). Nickel-chromium-molybdenum alloys and titanium have not been reported as being susceptible to MIC under field conditions, at least based on the literature review performed here. There is, however, one exception to this in environments containing oxygen. In surface waters and sediments containing oxygen, several microorganisms can oxidize dissolved manganese to form enriched mineral-biopolymer deposits. Deposits of manganese oxides, when formed on SS and CRA, are highly cathodic and result in localized potential differences that can drive severe corrosion (Lewandowski and Hamilton 2002). These deposits can be thin and brittle, resulting in fine cracks in the scale that act as crevices where corrosion is driven by the large corrosion potentials (E_corr_) between manganese oxides and the exposed metal. Although the corrosion in this example is not directly caused by microorganisms, the mineral scales resulting from their activity resulted in localized corrosion by shifting E_corr_.

Copper-nickel and nickel-based alloys have been used successfully in flowing, aerated seawater service, although MIC has been reported in some cases, particularly where flow is stopped for extended periods of time (Javed et al. 2016a). One study (Little et al. 1990) discussed severe corrosion of copper-nickel (88.5% copper, 10% nickel, and 1.5% iron) piping after 1 year of service and nickel alloy (66.5% nickel, 31.5% copper, and 1.25% iron) after six months of service in stagnant estuarine water from the Gulf of Mexico. In both cases, localized corrosion was found under biofilms containing SRB.

The susceptibility of different materials to MIC has been investigated by many researchers under laboratory conditions. Javed et al. (2020) reviewed 26 papers where MIC pitting was claimed to have been observed in laboratory tests on SS alloys, including 304, 316, 2205, and other alloys. The work concluded that the pits that formed as a result of the dissolution of inclusions (during cleaning) were comparable in shape, size, and depth to the pits that have been reported (possibly incorrectly) in the literature as indications that MIC attack had taken place on SSs. In another study, Javed et al. (2016b) demonstrated that the chemical composition and microstructure of different grades of CS influenced initial bacterial attachment and subsequent corrosion in the presence of *E. coli*. The work showed that the number of attached bacterial cells was different for different grades of CS and decreased with increasing pearlite phase content of the CS.

Another topic worth noting is the potential for metallurgical features such as inclusion content and surface roughness to affect biofilm establishment and corrosion rates. One industry study (Blythe and Gauger 2000) of welded CS found that:

- No correlations were found regarding the effects of surface finish on the severity of MIC and the relationship between colonization versus the inclusion content and composition of the steels tested.
- Steels with lower inclusion content and fewer sulfide inclusions consistently showed lower corrosion rates in the testing, even though colonization was similar to other steels.
- Microorganisms did NOT preferentially attack MnS inclusions in the test.
- SRB were not required to cause MIC, although they increased the severity of the attack.

Other work, however, has indicated a link between the location of manganese sulfide inclusions in CS and localized pitting attack when samples were exposed to SRB (Avci et al. 2013, Avci et al. 2018).

While the use of CRA with a high resistance to localized pitting is a possible approach to help avoid MIC, it is not economical in most cases. As a result, most oil and gas operations rely on CS as the primary material of construction. Some advantage can be gained, however, in selectively applying CRA where the threat of MIC is the highest. It is fairly well established, e.g. that areas of dead legs in piping are more susceptible to MIC than pipeline sections that normally experience flow. A number of schemes for assessing and ranking the threat of MIC have been published (Wolodko et al. 2018). The threat of MIC in dead legs can be managed by material selection in the design stage, retrofitting CS with CRA, or eliminating the environment that promotes MIC. Produced water, seawater, and firewater systems are also highly susceptible to MIC. Non-metallic components (i.e. epoxy composite piping, etc.) can be considered where pressures, stresses, and fire resistance requirements allow alternatives to metals. Above-ground piping for saltwater disposal systems, e.g. is sometimes constructed using fiber-reinforced plastic (FRP).

The application of CRA in equipment or piping that is highly susceptible to MIC can be made more economical using CRA-clad CS or limiting the use of CRA to only the most susceptible locations that cannot be temporarily isolated, cleaned, and chemically treated. Limiting the extent of MIC-susceptible equipment that cannot be cleaned, flushed, and treated is another way to reduce the need for CRA.

Internal coatings and linings of CS equipment are other approaches that can be used to avoid contact between the environment and material, at least for a finite period, i.e. the life of the coating. The use of high-density polyethylene liners in short sections of piping may also be a viable alternative. Potential issues with internal coatings and linings are damage from heat or rapid depressurization, mechanical damage during operation or maintenance, the absence of coating on tie-in welds, and a lack of insight for inspection site selection due to the presence of coating.

### Welds and MIC

One of the well-documented failure modes for MIC is the rapid attack of weld regions, with widespread reports of through-thickness pitting in the timescale of months. There are many examples of such failures, which often manifest as small pinholes on the surface with a large cavity in the weld region underneath, e.g. (Kearns and Borenstein 1991, Borenstein 1991a, 1991b, Jenkins and Doman 1993, Kobrin et al. 1997, Borenstein and Lindsay 2002). While some early reports suggested that the associated surface morphology may have been unique to MIC and hence a way of diagnosing the failure cause, other work has shown that similar surface pitting can be observed for non-biological corrosion (Thomas and Chung 1999). Problems have been reported with welds of different metal types, including SS, CS, and aluminum (Walsh 1999a). A number of causes have been attributed to the accelerated corrosion of welds, including associated microstructure (Walsh et al. 1993, Sreekumari et al. 2001) and composition (Walsh 1999b, Shi et al. 2020), while there is some debate about how/whether surface roughness might be involved (Walsh 1999a, Sreekumari et al. 2001, Amaya et al. 2002, Liduino et al. 2018). The microorganisms most associated with weld MIC are metal-oxidizing bacteria and SRB (Licina and Cubicciotti 1989, Ray et al. 2010, Liduino et al. 2018, Lee and Little 2019). There have been some reports that weld post treatment, including annealing and avoiding/removal of heat-tinted scale (e.g. gas shielding during welding and pickling), can help to reduce these problems (Stein 1991, Borenstein 1991a, Pytlewski et al. 2001, Davis 2006, Ehrnstén et al. 2019). While these measures may help avoid MIC problems, it is important to note that there can be some practical difficulties in implementation (Hurh et al. 1999, Ehrnstén et al. 2019).

Lastly, there are a number of important points to remember in relation to metals when performing laboratory studies of MIC. As discussed above, there are numerous factors that can affect the likelihood and extent of MIC for a particular metal type. This includes (but is not limited to) surface finish, specific chemical composition, and microstructure. Researchers should be conscious of these factors and make sure that they design tests accordingly and provide detailed information on these aspects so that the tests can be compared appropriately and are repeatable. A list of examples of techniques that can be used to provide important information on metallurgically relevant properties related to MIC studies is provided in Table 5.

**Table 5.** Examples of analytical techniques for the study of metal surfaces and corrosion by-products.

Analysis techniques	Description	Pros	Cons	References
Mass loss	Measuring the change of mass of a test coupon provides an indication of corrosion rate.	Relatively simple.	Assumes general / uniform corrosion. Localized corrosion often more important for MIC.	(ASTM G1-03 2017)
Surface profiling	Provides information on localized corrosion rates and morphology. Various methods (e.g. AFM, 2D and 3D scanners, manual pit gauge).	Localized corrosion attack common for MIC. Manual pit gauges are relatively simple and cheap, good if pits are relatively deep (i.e. ⪆ 100 µm).	Some of these techniques can be time-consuming and/or expensive equipment required. Manual pit gauge might not have required resolution in some cases.	(ASTM G46 - 94 2018)
Scanning electron microscopy (SEM)	Provides information on localized corrosion morphology.	Quick, widely available, may require sample to be vacuum compatible.	Morphology cannot necessarily be directly related to MIC attack.	(Dang et al. 2022)
Energy-dispersive X-ray spectroscopy (EDS)	Often combined with SEM. Provides spatially resolved elemental analysis, useful for determining corrosion products.	Quick, widely available.	Semi quantitative, numerous elemental peaks overlap, less information than other surface analysis methods.	(Ray 2010)
X-ray diffraction spectroscopy (XRD)	Technique for characterizing crystalline materials, useful for determining corrosion products.	Minimal sample preparation. Can identify key MIC by-products.	Cannot identify amorphous materials.	(Little et al. 2015)
X-ray photoelectron spectroscopy (XPS)	Based on the photoelectric effect, identifies elements present on the surface of a sample, useful for determining corrosion products.	Provides detailed chemical state information, quantitative analysis.	Not commonly available, sample must be ultra-high vacuum compatible.	(Kearns et al. 1992)
micro-Raman spectroscopy (Raman microscopy)	The pairing of the Raman spectrometer with an optical microscope (e.g. confocal Raman microscopy – CRM) allows to characterize the composition and structural organization of corrosion products.	Non-destructive; fast acquisition time.	Cannot detect zero-valent metal, only oxidized or reduced metal species	(Colomban 2011; Lanneluc et al. 2015; Refait et al. 2020; Trif et al. 2018)
FTIR (Fourier transform infrared) spectroscopy / ATR (Attenuated total reflectance)-FTIR	Allows the identification of chemical functional groups, able to differentiate organic and/or inorganic materials and microorganisms present on the surface of a sample, thus being useful to identify corrosion products, or monitor microorganism-surface interactions.	Quick, non-destructive, widely available, minimal sample preparation to obtain chemical complex information.	Can only be used in a laboratory setting and is sensitive to water, which can mask and disturb the obtained spectra. There is no distinction between the viable and dead cells.	(Basera et al. 2019; Bremer and Geesey 1991; Romero et al. 2007; Welikala et al. 2022)-Test

As discussed earlier, MLOE is required (microbiological, metallurgical, and media chemistry) to be able to distinguish between MIC and abiotic corrosion. There are no specific rules about which exact analysis methods need to be used, and will likely depend upon what methods/instruments are available, costs, and any specific information needed that might be related to particular corrosion processes of interest. In general, the use of multiple techniques to analyze each of the microbiological, metallurgical, and chemistry aspects can be beneficial; however, care and skill are needed to ensure that each test type is performed and analyzed correctly. Finally, control tests should be considered as a baseline comparison where possible. For example, it is critical to perform the same tests for a site with similar environmental conditions that has no signs of MIC as the location where MIC is suspected.

### Silos—overcoming barriers to interdisciplinarity in MIC studies

There are a number of barriers that make achieving true interdisciplinarity in MIC studies a challenge. As described earlier, each discipline typically exists in a relatively siloed environment where other disciplines are acknowledged but with whom regular dialog is relatively limited. Each discipline has its own unique language and worldview, which complicates translation between different disciplines. Even different sectors within a discipline may exist in silos, e.g. microbiology in human health vs. microbiology in industrial settings. For example, there has been very little translation of learnings from the biodeterioration of medical implants to microbial corrosion under non-medical conditions. Different disciplines and sectors also have different motivators and available resources that drive research and collaboration. On the industrial side, e.g. oilfield microbiological research around souring and corrosion has historically received much greater financial support than microbiological issues in, e.g. the pulp and paper industry. Further, in terms of motivation to support research, industry may be more concerned about protecting trade secrets and maintaining positive public perception than sharing detailed information about MIC issues and shortcomings in their mitigation programs. Likewise, academia’s motivations for participation in research include the continued need for peer-reviewed publication, requirements for bringing funding to a department, and the support of work for graduate students. These are examples of only some of the silos that challenge multidisciplinary work. The need for multiple disciplines to understand MIC is where the roles of chemistry, microbiology, metallurgy, physics, electrochemistry, genetics, and other sciences are essential for understanding how the overall environment can result in MIC.

A case study by Dubilier et al. (2015) discussed a global effort by scientists studying the Earth’s microbiome, where after ten years of work it was found that most of the data collected from different labs were not comparable because of differences in the test platforms used, the PCR primers selected, reporting formats, etc. This demonstrates that even for high-priority projects with a great deal of potential to improve human health, there is a great challenge to get all the various participants on the same page to achieve a successful conclusion. Ledford (2015) discussed one cause for the general lack of interdisciplinarity as being organizations’ “underestimating the depth of commitment and personal relationships needed for a successful interdisciplinary project.” It is likely that anyone who has experienced research projects that were run successfully and collaboratively can identify a core group of leaders in the project who promoted open technical exchange and worked well together as a team because of their personal commitment and value placed on relationships. Advancing interdisciplinary collaboration in the area of MIC will be essential to future progress in managing this integrity threat and increasing the sustainability of assets, particularly as used in renewable energy production.

## Laboratory models for microbial corrosion studies

**Figure 2.**
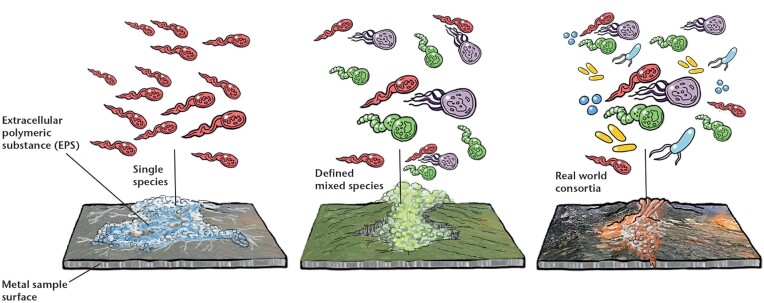
Depiction of the potential increasing complexity of different combinations of microorganisms in model systems that can be used to study MIC. Note that the depiction of the EPS and metal surface changes is not intended to indicate how the different model microbial systems affect corrosion outcomes.

MIC has been studied for over a century, with an explosion of publications emerging in the past 20 years (Lekbach et al. 2021). As microorganisms are essentially everywhere, including associated with man-made infrastructure, MIC has been studied across many sectors that include marine systems (e.g. shipping and marine infrastructure), energy systems (e.g. oil and gas), and in both domestic and industrial water and wastewater systems. As such, different models have been used for studying MIC and its potential threat to infrastructure (Fig. 2). It must be noted that the outcome of tests with such models will be influenced by multiple factors related to the test set-up and microorganisms used; as indicated in several sections above, the microorganisms, metal types, chemical environments, and operating conditions will affect whether MIC occurs. The effects of experimental conditions have been discussed by a number of authors previously (e.g. Wade et al. 2017, Salgar-Chaparro et al. 2020a,b). The focus of this section, however, is to review how the choice of microorganism(s) used may influence MIC tests.

By far, most laboratory-based MIC studies have used pure cultures of microorganisms (Lekbach et al. 2021), but more and more studies are emerging wherein defined mixed cultures and complex field samples are also being studied to help ground-truth pure culture studies (Salgar-Chaparro et al. 2020a, Puentes-Cala et al. 2022, Sharma et al. 2022). Whether a pure culture, a defined mixed culture, or a complex model system is used to study MIC depends largely on the goals of the study. It must be emphasized that all approaches can yield valuable information but are also associated with limitations that should always be kept in mind when making conclusions about MIC.

### Single species models

A list of ∼50 different pure microorganisms associated with metal corrosion (primarily using CS or SS) was recently tabulated (Lekbach et al. 2021), and while many more are likely to be identified, it gives an indication of the diversity of taxa (both aerobic and anaerobic) that can be involved in MIC. For example, under aerobic conditions, *Pseudomonas* sp. has been studied the most frequently, while under anaerobic conditions, strains of sulfate-reducing microorganisms such as *Desulfovibrio* sp. have been the most widely used (Lekbach et al. 2021). While the major limitation in using pure cultures to study MIC is that they are not necessarily reflective of, nor participants in, real-world corrosion scenarios, studying MIC using pure organisms allows for highly controlled studies to better understand the behaviors and mechanisms of MIC. For example, experimental systems of any type (e.g. using EC techniques, bioreactors, weight loss experiments, etc.) can be established in the presence and absence of the pure culture of interest, and differences in metabolic indicators (such as electron donors and acceptors), EC signals, corrosion products, surface analyses, etc. can be determined between the live and control incubations (e.g. Tsurumaru et al. 2018, Tang et al. 2019, Lekbach et al. 2021 and references therein).

As discussed earlier, obtaining MLOE even in pure culture MIC studies helps to provide the strongest case of whether microorganisms contributed to a corrosion scenario. Notably, pure culture studies also allow for the simplest interpretations of any MMM that may be used to track microbial metabolism in a corrosion case, such as through transcriptomic, proteomic, or metabolomic approaches, again compared to a non-corrosion scenario. These types of approaches can potentially help to elucidate a target gene, protein, or metabolite that may be indicative of MIC. For example, if specific genes are upregulated during a corrosion versus a non-corrosion scenario, the expression of these genes may be important for MIC to occur. Ultimately, creating mutants wherein these genes are deleted and corrosion no longer occurs is a strategy that might be able to be used for linking specific genes/gene expression to MIC (Lekbach et al. 2021). For example, a gene deletion approach was used to help provide evidence that a corrosive methanogen (*Methanococcus maripaludis* strain OS7) uses an extracellular [NiFe] hydrogenase in MIC (Tsurumaru et al. 2018). Subsequently, a qPCR assay was developed to quantify this gene (*micH*), which could be detected in corrosive but not in non-corrosive biofilms established from oil field samples (Lahme et al. 2021). A gene deletion approach was also used to help pinpoint that *Geobacter sulfurreducens* could corrode Fe^0^ by using it as a sole electron donor (Tang et al. 2019), as well as to suggest that *Shewanella oneidensis* strain MR-1 can corrode CS both directly and through hydrogen-mediated electron transfer (Hernández-Santana et al. 2022).

### Defined mixed species

In the real world, microorganisms exist in most environments in the form of complex multispecies consortia. MIC can occur due to both planktonic and surface-attached microorganisms and their metabolic by-products. The compositions of the microbial consortia in a particular location will be affected by a variety of biotic and abiotic parameters (e.g. temperature and other physicochemical properties, nutrient supply, fluid mixing, etc.) (Fuhrman et al. 2015, Dang and Lovell 2016). In relation to the attached/biofilm versions of microbial consortia, it is generally acknowledged that the microorganisms attach and form a biofilm in a sequence and that the creation of a biofilm can offer overall benefits to the community, such as enhanced resistance to stress and disinfectants (Bridier et al. 2011, Schwering et al. 2013, Burmølle et al. 2014). The presence of different microbial species in a consortium can lead to interspecies cooperation where, e.g. certain species may provide nutrients or create habitats that are essential for other species. In relation to MIC, aerobic biofilm formers may attach early and create anaerobic niches that are suitable for anaerobic species (such as *Desulfovibrio* sp.) that have been implicated in accelerated corrosion. Multispecies models have been developed to simulate environments such as oral biofilms (Kommerein et al. 2018); however, there are many challenges involved, such as determining which species/characteristics should be included, the order of inoculation and nutrients, and other environmental conditions (e.g. flow and redox poising) (Foster and Kolenbrander 2004, Røder et al. 2016, Tan et al. 2017, Olsen et al. 2019). There has been some work performed on defined multispecies models for MIC studies, but aside from a few cases, it has typically been limited to combinations of two bacterial species (Phan et al. 2021, and references therein). This is an understudied area with potential for much future research to better understand the fundamental processes involved in MIC when more than one microorganism is present and to produce multi-species models that better simulate the rates and types of accelerated microbial corrosion observed in the field.

### Real-world consortia

The final type of model system that can be used to study MIC is one that uses samples taken, or continuously sampled, from the field as the test medium or as inoculant for the test system. This approach can provide conditions most closely representing the real world. An example of this is the work of Lee et al. (2004), where natural seawater was used as the test medium for an MIC study. Changing the test conditions in this example system (creating a stagnant anaerobic solution) resulted in increased numbers of SRB present and led to more aggressive corrosion. In another example, Marty et al. (2014) reported a corrosion test reactor system that utilized natural marine microbial consortia, was capable of simulating tidal changes, and was able to supply a continuous flow through the test system of natural seawater. Changes in test conditions (e.g. providing an initial pulse of organic matter) were shown to lead to increases in localized corrosion rates, and the identification of similar bacterial populations to those identified in accelerated low-water corrosion suggests that the system can be used to simulate real-world marine conditions. In another example, Wade and Blackall (2018) used samples of accelerated low-water corrosion products as the microbial inoculum in corrosion tests and varied the testing conditions. The results obtained showed how changing the specific test conditions (e.g. by adding nutrients) can affect both the magnitude of corrosion that takes place and the microbial community that develops. A key issue from these types of studies is that taking the microbial samples out of the field changes the environmental conditions and hence affects the test outcome in some way. Salgar-Chaparro et al. (2020b) showed for tests using microbial consortia sampled from floating production storage and offloading facilities that changes in supplied nutrients affected biofilm properties and subsequent corrosion. Studies using real-world consortia with minimal alteration have the least control over the specific microbial species present and suffer from increased difficulties in terms of reproducibility. Additional studies that minimally alter the conditions of the samples being tested (e.g. by avoiding nutrient additions or changing the water-to-solids/biofilm ratio) are also needed to help better understand MIC under realistic, real-world conditions in multiple environments (Wade et al. 2017).

Laboratory models are an integral part of the overall efforts to tackle the challenges associated with MIC. They can provide key information on critical aspects such as the fundamental processes and microorganisms involved, the performance of materials and mitigation methods, as well as a means for MIC diagnosis. Microbiologists are well placed to offer leadership and guidance on many facets of future MIC laboratory model development.

### Field (meta)data collection and standardization

To date, there has been limited success on predicting MIC problems and evaluating potential mitigation strategies. Significantly more work will be required to achieve effective and tailored anti-MIC measures. An example of one of the key challenges that remains to be addressed and overcome is the lack of readily available key data repositories, i.e. field (meta)data collections relevant to industrial applications, such as biobanks of biofilm samples and MIC samples (e.g. materials with MIC, environmental, and metallurgical data). This requires the development of standard data collection and assessment protocols to ensure consistency and allow appropriate analysis and comparisons to be made. Such repositories are critical for increasing knowledge and promoting new advances in the field, which potentially may be enhanced by integrating artificial intelligence and machine learning techniques (Goodswen et al. 2021). Such tools are essential for modeling and predicting MIC scenarios, discovering MIC markers and biosensors, and developing standards.

In this context, developing standardization of measurement procedures, relevant protocols (e.g. sample preservation), validation tests, and methodologies is an essential step towards improved MIC mitigation. Standardization helps to ensure that MIC-related assessment tests are accurately cataloged, allowing them to become comparable or able to be correlated, thus leading to a more comprehensive understanding of MIC and MIC control strategies. Unfortunately, gaps in the field continue to delay the development of universal standards. For example, there is still a significant lack of translation of small-scale research laboratory experiments to a field scale. Likewise, there have been only limited efforts to develop well-validated models (physical and theoretical) that simulate the complex real-world conditions. These efforts are essential tools for standardization and the development and assessment of mitigation solutions, which could save time and resources before the final validation stage. Further work is also required to develop standards relevant to or adopted by legislation or regulatory assessment (e.g. standards to assess the efficiency and effectiveness of biocidal mitigation strategies) that more closely match real-world conditions. Efforts have been made in specific fields to overcome this gap (Skovhus 2014, Silva et al. 2021), particularly with the introduction of MMM (Skovhus 2014). Even so, most MIC researchers use protocols or methodologies adapted from inaccessible or expensive organizational standards (e.g. ISO, ASTM, and NACE) to evaluate their approaches or technologies, whereas industry uses the available organizational standards or develops its own (Skovhus et al. 2017, Silva et al. 2019, Wade et al. 2023).

## Corrosion management

**Figure 3.**
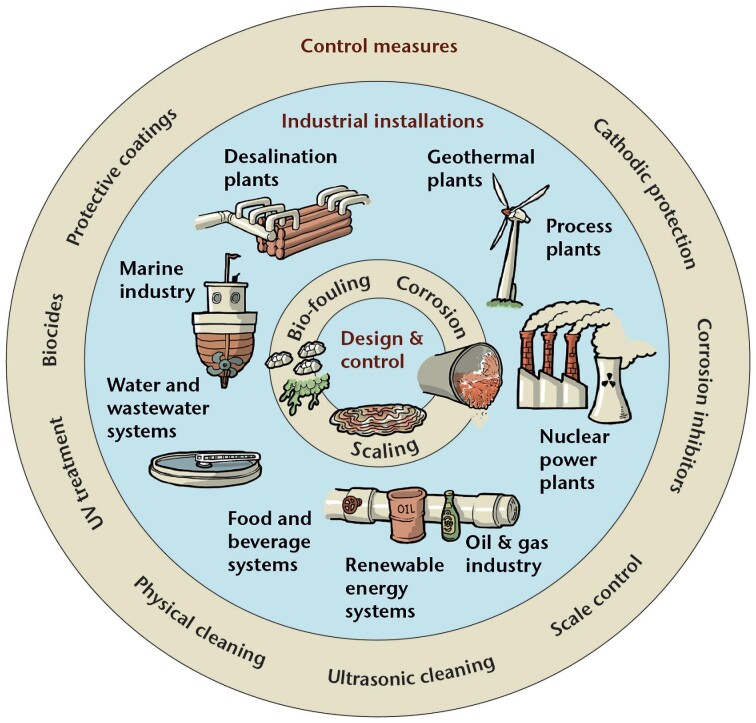
Examples of different strategies used for MIC control.

MIC is regarded as a difficult-to-treat industrial “cancer” (World Corrosion Organization (WCO) Shenyang Declaration, 2019), resulting in severe economic losses and underestimating long-term environmental and societal impacts (Usher et al. 2014, Conley et al. 2016, Di Pippo et al. 2018, Jia et al. 2019, Stamps et al. 2020, Little et al. 2020b, Lou et al. 2021). It has undoubtedly become vital to not only understand the MIC phenomenon but also how to control it effectively. To date, a range of methodologies and technologies have been designed, developed, and implemented to control microbial activity and thus reduce the threat of MIC (Fig. 3). The characteristics of each system and field environment will dictate the selection of a specific countermeasure, whether based on removal and/or preventive strategies.

In industry, the corrosion control process typically consists of three primary activities: (1) identifying the relevant corrosion threats; (2) identifying preventive and mitigative measures to address those threats; and (3) monitoring the effectiveness of the response. The cycle of activities is continuous, with each of the three activities providing input to the subsequent activity. Information about a system’s microbiology is typically needed in each of the three corrosion control activities, and MMM is increasingly being used to provide that information. However, corrosion engineers also need a way to correlate microbiological information with other relevant information, such as data from corrosion monitoring (e.g. coupons, probes, and inspection), operating conditions (e.g. pressure, temperature, and fluid velocity), fluid composition and chemistry, mitigation measures, etc. Such an approach is consistent with the use of MLOE, as described earlier in this review. The following section briefly describes each of the three corrosion management activities that are employed to manage internal corrosion on various types of assets in different sectors.

### Threat assessment

During the corrosion threat assessment stage, the potential for each plausible corrosion threat mechanism is evaluated. Corrosion engineers typically review data about the asset design and overall process, operation, chemical treatment, corrosion monitoring data, and leak/failure history data to help identify corrosion threats. The potential damage rate of some threats, such as corrosion caused by acid gases, can be estimated using mathematical models; however, there are presently no widely accepted corrosion rate models for MIC since microorganisms can influence corrosion in many different ways. In assessing the potential for MIC, the corrosion engineer typically looks for a relationship between the microbiological and chemical conditions and any observed corrosion information. Data produced using MMM are used in this step to characterize baseline microbiological conditions in the asset and to look for associations between biofilm community distribution, chemical composition, and the frequency, distribution, and severity of localized corrosion. The threat assessment may also seek to relate biofilm and corrosion characteristics to operating conditions, such as changes in flow (e.g. periods of no flow), temperature, or fluid composition (e.g. increases in nutrients or electron acceptors). Significant operating condition changes may affect the initiation and/or propagation of MIC. A number of investigators, such as Skovhus et al. (2010), Eckert et al. (2012), and Larsen and Hilbert (2014), have demonstrated the utility of MMM in forensic corrosion investigation, where methods such as next-generation sequencing and metagenomics could provide insights.

### Mitigation and prevention

Based on the threat assessment, the preventive and mitigative measures needed to manage the applicable corrosion threats are selected. Options for internal corrosion mitigation in pipelines include the use of biocides, corrosion inhibitors, or oxygen scavengers; velocity control; mechanical cleaning (e.g. pigging or flushing); ultraviolet radiation; fluid process vessels (e.g. filters, separators, etc.); or control of fluid quality (or sources) to the extent possible. Larsen et al. (2010) demonstrated how MMM were beneficial for evaluating the effectiveness of new chemical treatments when corrosion incidence rate and severity are linked with observations about the types, numbers, and activities of microorganisms after the treatment is applied. One of the most significant challenges to this process is the collection of biofilm samples from the asset being treated and the processing/analyzing the samples in a timely manner so that genetic information is not lost. Another significant challenge is the current lack of standards to assess the efficiency and effectiveness of MIC mitigation strategies based on microorganisms in biofilms or MIC diagnosis and monitoring methodologies, as the conditions promoting MIC may be quite different from system to system.

In terms of MIC mitigation, most conventional strategies comprise physical and/or chemical methods. Mechanical removal or cleaning of surfaces is the most straightforward physical approach, comprising any method able to remove the biofilm attached on a surface, involving those using mechanical forces (e.g. pigging, flushing, ultrasonic treatment). However, this is not the optimal approach for MIC control as it does not prevent further biofilm formation, demanding costly ongoing maintenance and retrofitting measures. For example, once a surface is in contact with seawater, a biofilm can form in minutes and progress to macrofouling in just a few days, which would require frequent maintenance, rendering it an unsustainable mitigation strategy (Omar et al. 2021, Silva et al. 2021, Yazdi et al. 2022).

The most effective countermeasures currently adopted to control biofilm development and minimize MIC on industrial surfaces rely on a chemical strategy that comprises the direct or controlled release of biocides onto the contaminated surface. Their use is promoted on the basis that disinfection, or killing microbial cells, will solve the problem. However, inefficient cleaning of organic matter remaining on the surface and inadequate monitoring strategies, allied to a lack of skilled MIC professionals, can actually promote an increase of MIC problems. Thus, chemical strategies are generally integrated with other methods, such as protective polymeric coatings, cathodic protection (CP), UV irradiation, mechanical cleaning, or ultrasonic treatment. Among those, antifouling coatings containing active agents, i.e. biocides and corrosion inhibitors are one of the most well-established preventive measures (Abdolahi et al. 2014, Cai et al. 2020a, Chen et al. 2022, Lamin et al. 2022, Wen and Li 2022). A significant disadvantage of these coatings, however, is the continuous release of toxic and persistent chemicals, resulting in shorter protection periods and potential ecological problems (Rosenberg et al. 2019, Mansor and Tay 2020, Machate et al. 2021, de Campos et al. 2022). Their use is now strictly regulated in certain areas [e.g. EU Biocides Regulation 528/2012 (98/8/EC)], prompting the search for effective and environmentally friendly long-term solutions (Loureiro et al. 2018, Ferreira et al. 2020, Vilas-Boas et al. 2020, Ferreira et al. 2021). Significant advances have been achieved in relevant coating technologies, with work on a range of different properties such as the group of targeted organisms, mechanism of action, bioactive agents, the polymeric matrix, surface structure, environmental surrounding, or even fundamental working principle being conducted (Ferreira et al. 2020, Bhoj et al. 2021, Silva et al. 2021).

Other greener or less toxic alternatives with enhanced effects have also emerged. From coating strategies based on the development of polymer structures to create or improve properties such as hydrophilicity, amphiphilicity, surface topography, non-biocide-release mechanisms and/or the incorporation of bioactive nanoparticles to generate nanocomposite coatings (Selim et al. 2020; Gu et al. 2020; Kumar et al. 2021; Sousa-Cardoso et al. 2022), to the search for nature-inspired biomimetic and synthetic agents to natural bioactive compounds or extracts (e.g. metabolites from marine organisms, molecules of microbial origin, plants) (Vilas-Boas et al. 2021; Lavanya 2021). However, the full exploitation of these greener agents is limited by long synthesis processes, low yields, the scarce availability of some natural sources, the lack of proof of concept in real-world conditions, the absence of an environmental impact assessment, as well as the need for significant funding and time for approval by regulatory agencies (Qian et al. 2009, Brinch et al. 2016, Pai et al. 2022).

Another of the commonly discussed methods for MIC mitigation, used for a range of structures such as buried and submerged pipelines, storage tanks, and sheet piling, is the application of CP (Wilson and Jack 2017, Ackland and Dylejko 2019, Angst 2019). This technique involves the application of a direct current (via a galvanic or impressed current system) to lower and maintain the potential of the metal sufficiently negative with respect to the environment. CP is a well-known and widely applied method for abiotic corrosion, and it is often discussed that a further lowering of the protection potential from that used for abiotic corrosion may provide protection against MIC. While field tests and anecdotal reports indicate CP may be capable of preventing accelerated corrosion due to microorganisms, the conclusions of laboratory studies are much less certain, and there is room for much more work on this topic to understand the mechanisms involved and how to optimize its use for avoiding/minimizing MIC (Thompson et al. 2022).

### MIC mitigation challenges

**Figure 4.**
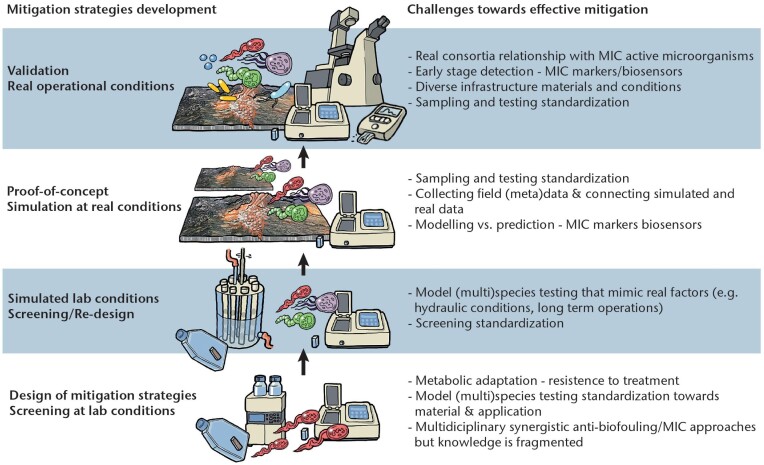
The main steps involved in developing an MIC mitigation strategy, along with associated challenges to attain effective solutions.

Similar to MIC research in general, mitigation strategy development is also greatly affected from the initial design stage to the final implementation by the siloed nature of this field. Figure 4 summarizes and highlights some of the most important challenges that need to be overcome in order to allow successful MIC mitigation strategy development and implementation. To achieve this, the following key questions need to be answered:

- What are the challenges/knowledge gaps to control MIC?
- What are the current needs for the development/implementation of anti-MIC strategies?
- What tests and metrics are appropriate to evaluate the effectiveness of an anti-MIC strategy?

### Understanding the biofilm community interactions with the environment and surfaces

Biofilm and subsequent MIC are driven by environmental conditions, either natural or under industrial operating conditions, involving ecological and engineering factors. Understanding and identifying the role of microorganisms in MIC is a big challenge, as the composition of the biofilm matrix and its dynamic structure will vary depending on those conditions, and contact with different infrastructure materials, resulting in metabolic adaptations in response to their long-term survival under external stress conditions (Jia et al. 2019). Cells incorporated within a biofilm, e.g. show a high tolerance to treatment compared to planktonic cells. In an extreme scenario, this can result in an increase of resistance to antimicrobial agents of 1000 times (Mah et al. 2003). It is also recognized that the response of the biofilm community to environmental conditions cannot be predicted by studying free-living bacteria or single-species biofilms alone (Flemming et al. 2016). For the initial step of designing a mitigation strategy, these studies are nevertheless useful for a screening task, but multi-species studies are even more critical to improve the design. This fundamental understanding of the complex properties of biofilm communities and their interaction/development with different environments, including biota and conditions fluctuations from static and quasi-static to dynamic flow conditions (Toyofuku et al. 2016), remains limited, and further advances in the design of effective mitigation countermeasures are desired. This progress is hampered further by a lack of understanding of surface-biofilm interactions and their heterogeneity, which promote localized gradients and microenvironments across the surface (Ren et al. 2018) and may involve multiple microbial mechanisms.

Understanding how surface and biofilm structures and their physicochemical properties interact, e.g. provide answers on which factors contribute to biofilm structure and composition and how the multi-species system interacts is critical for developing a better strategy against MIC and avoiding the implementation of mitigation actions when they are not needed (Skovhus et al. 2022).

### Limited and fragmented knowledge on mitigation strategies

Problems due to the resistance of biofilms to treatment can hamper the effectiveness of mitigation strategies, particularly those involving the release of active agents such as corrosion inhibitors and bioactive agents (e.g. biocides and biocide-release coatings). Biofilm resistance is related to the complex three-dimensional functional structure biofilms, which limits the penetration of bioactive agents and prevents them from interacting with other cells, particularly for mature biofilms (Bas et al. 2017, Merchel Piovesan Pereira et al. 2021). This is complicated by the complex processes by which bioactive agents interact with biofilms, involving biological and physicochemical factors, and the exact degree, frequency, and mechanisms that give rise to resistance are still unclear.

Bioactive agents are commonly selected based on the following criteria: the spectrum of action/efficacy, toxicity, biodegradability, cost-effectiveness, environment safety, and compatibility with the system, i.e. allow the maintenance of fluids and materials under operational conditions. Furthermore, the mode of action depends on the type and dose of bioactive agent used (Sharma et al. 2018, Capita et al. 2019). However, their long-term use can promote the resistance of microorganisms, leading to an ineffective inhibition effect.

The use of corrosion inhibitors is another simple and potentially efficient mitigation strategy. A diverse range of chemical molecules acting as corrosion inhibitors has been exploited, mainly including surfactants and heterocyclic organic compounds containing electrically rich heteroatoms (N, O, and S) or groups with π-shared electrons (Feng et al. 2022). Those primarily inhibit corrosion by adsorbing on metal surfaces by physical adsorption (Vander Waals force adsorption) or chemisorption (chemical bonding), creating a physical or chemical barrier between the surface and the corrosive media, hence inhibiting cell adhesion and subsequent biofilm formation (Migahed and Al-Sabagh 2009, Kokalj 2022, Ma et al. 2022).

Combining corrosion inhibitors with bioactive agents has also been a common strategy to find synergistic effects (Greene et al. 2006, Pinnock et al. 2018, Anandkumar et al. 2023). However, it can sometimes lead to interferences affecting agents’ performance, such as chemical incompatibility (e.g. chemical and physical interactions, pH range of action) and competitive function (e.g. adsorption for the same metal sites), reducing their primary function and resulting in inadequate control of MIC (Maruthamuthu et al. 2000, Xiong et al. 2015, Rahmani et al. 2016). To avoid interferences, these agents need to be carefully selected, not only considering standard criteria like the ability to oxidize the metal, the presence of a particular functional group, the capacity to cover a wide area, cost-effectiveness, solubility, and environmental safety (Lavanya 2021), but also the conditions present throughout the entire system.

Certain corrosion inhibitors can also interact with biofilms and impair their structure and functionality. For example, positively charged heterocyclic quaternary ammonium salt surfactant can selectively adsorb on the negatively charged SRB biofilm surface and penetrate the cell membrane, disrupting their selective permeability and genetic system, thus leading to the inhibition of SRB activity or death (Feng et al. 2022). This shows the ability of synthetic corrosion inhibitors to also provide antimicrobial effects. This multifunctional ability has been reported particularly for cationic surfactants, including Gemini and poly (quaternary ammonium) salt surfactants (Badawi et al. 2010, Labena et al. 2020, Feng et al. 2022). The effectiveness of these mechanisms, however, depends on the specific system’s conditions and the microorganisms involved. In some cases, they may even become ineffective (Dariva and Galio 2014, Mand and Enning 2021) or act as a source of nutrients for bacterial growth (Edwards and McNeill 2002, Fang et al. 2009) Therefore, it is crucial to fill the knowledge gap regarding the mechanisms of action of corrosion inhibitors and bioactive agents, as well as their effects on the development and resistance of biofilms (Bridier et al. 2011, Bas et al. 2017, Kimbell et al. 2020, Tuck et al. 2022). Despite progress over recent years, knowledge is still scarce and fragmented (Araújo et al. 2014, Huang et al. 2020, Silva et al. 2021, Lima et al. 2022).

Furthermore, similarly to bioactive agents, the long-term use and toxic characteristics of synthetic corrosion inhibitors calls for more work on sustainable and environmentally friendly agents derived primarily from natural sources (Lavanya 2021, Verma et al. 2021, Al Jahdaly et al. 2022, Fazal et al. 2022, Wang et al. 2023a,b).

The increasing discovery of greener and natural agents, including corrosion inhibitors and bioactive agents, with new chemical structures and functionalities is likely to uncover additional modes of action (Lavanya 2021, Barba-Ostria et al. 2022). This is likely to further improve our understanding of the mode of action of bioactive agents and how they interact with biofilms, which is essential for developing more effective mitigation strategies. Artificial intelligence has been proposed as a potential tool to accelerate the identification of targets for novel active agents (Paul et al. 2021).

The growing cross-sectoral awareness of the economic importance of microbial biofilms has helped to accelerate the development of mitigation approaches and technologies as well as our understanding related to biofilm-bioactive agent interaction. However, some sectors possess more advanced knowledge than others. For example, the increasing problem of antibiotic resistance is well known in the healthcare sector, while the marine sector has been applying anti-biofouling approaches for some time. Regrettably, anti-biofouling, anti-MIC, or anti-corrosion approaches are rarely related, although a few recent publications have started to emphasize their similarities (Li and Ning 2019). This lack of sectoral and multidisciplinary knowledge sharing undoubtedly limits the potential for advances in mitigation strategies.

Finally, it is worth noting that the complete eradication of biofilms in most industrial situations is highly unlikely, as microorganisms will always be present. Thus, the economics and efforts required to meet such a stringent target need to be carefully questioned. A more realistic goal is to learn how to coexist with and manage the presence of biofilms, minimizing unwanted interferences in the most efficient, benign, and long-term manner possible. Furthermore, MIC management extends beyond single solutions. Rather, integrated approaches should be used, leveraging multidisciplinary teams and cross-sectoral knowledge sharing.

### Monitoring

**Figure 5.**
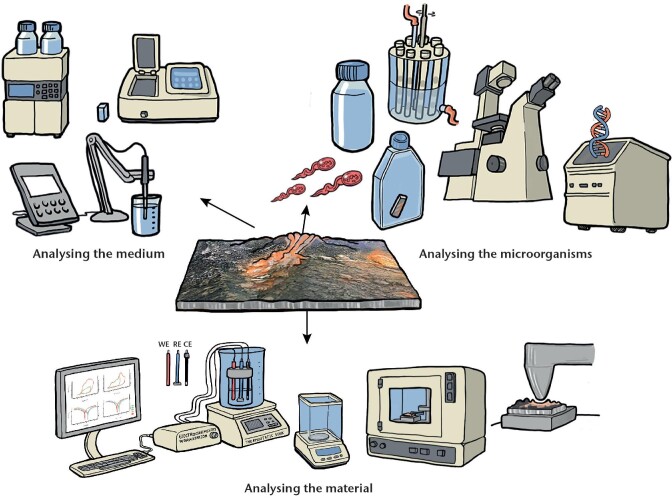
MIC monitoring requires integrative data analyses using a comprehensive range of tools.

The third core activity in the corrosion control process is measuring the system performance and effectiveness of the methods used to reduce the likelihood and/or severity of corrosion. Various corrosion monitoring techniques and inspection methods can provide information about the rate of metal loss due to corrosion (Bardal 2004, Dawson et al. 2010, NACE 2012); however, many of these methods do not identify the mechanism of the corrosion or the effects of mitigation activities on the cause of the corrosion, biofilms for MIC. Again, the MLOE approach is useful for evaluating the effectiveness of mitigation measures and optimizing these measures as system operating conditions and corrosion threats change over time (see Fig. 1). For MIC mitigation, monitoring measures ideally need to be able to identify both changes in corrosion rates and microbiological changes in associated biofilms (Fig. 5).

Corrosion engineers typically attempt to integrate MLOE, operating data, corrosion monitoring data, chemical/microbiological fluid and deposit analysis results, in-line inspection (ILI) and other inspection data, and flow/corrosion rate model outputs to ascertain the short-term and long-term effectiveness of mitigation measures. Short-term effectiveness (i.e. over hours, days, or weeks) may be evaluated through different parameters or measurements than those used to monitor long-term (i.e. monthly or annual) effectiveness. For example, short-term effectiveness monitoring could focus on controlling microbial populations in biofilms, whereas long-term effectiveness would focus more on controlling corrosion damage that resulted from those biofilms. One significant MIC management challenge faced by industry is the lack of an established, widely accepted processes (or standards) for integrating MLOE into monitoring programs. Often, microbiological data are incorporated into decision-making by inferring the activities and roles of microorganisms in corrosion mechanisms, and mitigation measures are adjusted based on empirical observations.

A key challenge in providing timely and effective anti-MIC measures is the establishment of early biofilm-specific detection systems suitable for in-situ and point-of-use industrial contexts (Xu et al. 2020). This could potentially include surface monitoring, regular chemical and microbiological analyses, and the use of probes, sensors, and MIC markers. Early prediction is critical during the initial and validation stages of mitigation strategy development (Fig. 4), as it allows the identification of specific locations with MIC threats as well as the evaluation, tailoring, and implementation of appropriate anti-MIC strategies.

Finally, one of the major problems with MIC prediction capacity is that the entire contextual story is not always reported or considered. For example, material engineers (e.g. metallurgists) may tend to ignore biological data, whereas microbiologists may not appropriately consider materials/metallurgical aspects. Collecting relevant data from all aspects (media conditions, fluids, microorganisms, and materials) requires multidisciplinary teams composed of operators, microbiologists, corrosion engineers, chemists, and materials engineers. Furthermore, for some, there has been a major stigma associated with revealing MIC cases. Hence, data on MIC failures in industry is largely inaccessible to the broader R + D community, while MIC mitigation business agents often protect customers through commercial confidentiality agreements, significantly limiting the availability of cases and the transfer of potential mitigation technologies between industry and academia. Development of forums for the sharing of MIC case histories with the appropriate level of detail so as to maintain the identities of those providing the information is one way to improve knowledge sharing.

## EC techniques used to study MIC

As noted earlier in this review, MIC is a multi-disciplinary field that requires expertise encompassing significantly different fields of study. The corrosion of metals (including MIC) is inherently an EC process where one or more chemical species undergo changes in oxidative state. Numerous EC techniques have been developed to mechanistically study fundamental corrosion mechanisms in the laboratory, in addition to monitoring corrosion behavior in field conditions. As most of these methods are out of the scope of expertise of many microbiologists, we decided to dedicate a separate section to provide some basic background information about EC methods and the main techniques used in MIC assessment. Nonetheless, the authors highly encourage the collaboration of microbiologists with subject matter experts in corrosion and electrochemistry. All EC techniques have limitations in application and interpretation; thus, the choice of technique and the interpretation of results need to be carefully weighted.

**Figure 6.**
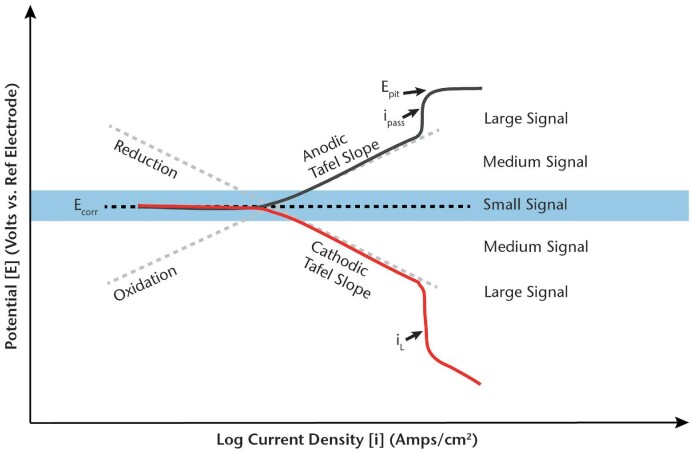
Key parameters associated with EC corrosion testing.

In the following sections, we aim to provide a more detailed overview about specific EC techniques that are beneficial during MIC studies. The EC techniques have been grouped based on the amount of external signal (e.g. applied potential or current) required during measurement. In general, the larger the external signal, the more information about the system can be obtained (Fig. 6); however, applying a larger signal can also result in alterations to attached biofilm or substrate surface chemistry. The traditional EC cell is a three-electrode system containing: (1) the metal of interest (working), (2) a stable (i.e. non-polarizable) electrode (reference), and (3) a corrosion-resistant metal used to complete the electrical circuit for external signal application (counter). Modifications on the number and types of electrodes are dependent on the EC technique being applied. The following is not an extensive review of EC techniques, rather those that are most commonly used in the design and monitoring of MIC experiments.

### Techniques requiring no external signal

The following techniques do not apply current or potential signals to the working electrode. These techniques are used to monitor corrosion behavior, but overinterpretation of the measurements is cautioned.

### Corrosion potential, E_corr_

The simplest EC technique is the potential measurement across a two-electrode system immersed in an electrolyte; where one electrode is the material of interest and the other is a stable reference electrode. The potential is measured across a high-impedance voltmeter that prevents current flow between the electrodes. This potential is called the E_corr_ but is also referred to as the open circuit potential. Regardless of nomenclature, E_corr_ measurement is a passive monitoring method that does not disturb an attached biofilm. Passive metals such as titanium and gold exhibit higher E_corr_ compared to more active metals such as zinc and aluminum. Biofilms can also affect E_corr_ and make the interpretation of results difficult (Little and Wagner 2001). The most common utilization of E_corr_ measurements has been the study of potential ennoblement. Ennoblement is the increase (i.e. more electropositive) of E_corr_ due to the formation of a biofilm on a metal surface (Little et al. 2013). Ennoblement of passive alloys exposed in marine environments due to biofilm formation has been extensively documented (Mollica and Trevis 1976, Johnsen and Bardal 1985, Scotto et al. 1985). Theoretically, E_corr_ ennoblement should increase the probability for pitting and crevice corrosion initiation and propagation for those passive alloys where E_corr_ is within a few hundred millivolts of the pitting potential (E_pit_) (Fig. 6). Little et al. (2008) reviewed mechanistic interpretations of ennoblement in marine waters. Ennoblement has also been shown to occur in fresh and estuarine waters through microbial manganese oxidation and deposition on the metal surface (Dickinson and Lewandowski 1996, Dickinson et al. 1996, Dexter et al. 2003). While the ennoblement phenomenon has been observed through the world under different water conditions, a unifying mechanistic explanation for all observations does not exist. The main drawback of E_corr_ measurement is the inability to interpret whether ennobled E_corr_ (or other changes in E_corr_) are due to thermodynamic effects, kinetic effects, or both. In addition, E_corr_ measurement alone cannot be used to determine changes in corrosion rates over time. Unfortunately, however, overinterpretation of E_corr_ with respect to corrosion rates is commonly found throughout the literature.

### Dual cell technique

The dual cell uses two similar EC cells that are separated by a semipermeable membrane. Each cell contains the same electrolyte and nominally similar working electrodes. The two working electrodes are connected electrically to a zero resistance ammeter (ZRA), and the semipermeable membrane provides ionic conduction to complete the circuit. One cell is maintained under sterile conditions. Microorganisms are added to the other, and the sign and magnitude of the resulting current through the ZRA are monitored to determine the details of the corrosive action of the bacteria. The dual cell technique does not provide a means to calculate corrosion rates, but rather changes due to the presence of a biofilm. Dexter and LaFontaine (1998) used a dual cell configuration to monitor the corrosion of copper, steel, 3003 aluminum, and zinc samples coupled to panels of highly alloyed SS. Natural marine microbial biofilms were allowed to form on the SS surface. On the control tests, the action of the biofilm was prevented. Corrosion of copper, steel, and aluminum anodes was significantly higher when connected to cathodes on which biofilms were allowed to grow naturally.

### Electrochemical noise analysis (ENA)

EC noise has conventionally been applied to two electrodes of the same material. ENA data can be obtained with applied signal (i.e. fluctuations of potential at an applied current or vice versa). In addition, ENA can also be operated with no applied signal, where small fluctuations of E_corr_ are recorded as a function of time. For MIC studies, the no-signal mode provides a monitoring technique that has a clear advantage over the applied signal mode that may influence biofilm properties. Under controlled laboratory studies, it is possible to measure potential and current fluctuations simultaneously. Simultaneous collection of potential and current data allows analysis in time and frequency domains. There are numerous parameters that can be determined through data analysis with EC noise resistance (R_n_) being the most commonly interpreted. To this day, the interpretation of R_n_ to a quantifiable corrosion rate is debated. Bertocci et al. (1997a,b) described methods for data analysis. Little et al. (1999) showed an example of using ENA to examine the influence of marine bacteria on localized corrosion of a coated steel. Samples with intentional defects in the coatings exposing bare metal were immersed in artificial and natural seawater with and without attached zinc coupons, providing sacrificial CP to the exposed areas. R_n_ increased with time for all cathodically protected samples due to the formation of calcareous deposits in the defects. Surface analysis showed that very few bacteria were present in the defects of the cathodically protected samples, while large amounts of bacteria were found in the rust layers of the freely corroding samples.

### Techniques requiring a small external signal

The following techniques require an external signal to be applied to the working electrode. There are, however, no standards in regards to the magnitude of the signal applied, which is most commonly an applied potential. The majority of applications of these techniques in the literature apply between +/-5 and 10 mV to the working electrode.

#### Polarization resistance technique

The polarization resistance (R_p_) technique is a direct current (i.e. no frequency dependence) method that can be used to continuously monitor the instantaneous corrosion rate of a metal, as detailed in ASTM G59-97 (2014) and reviewed by Scully (2000). Mansfeld (1976) described the use of the R_p_ technique for the measurement of corrosion currents.

A simplification of the R_p_ technique is the linear polarization technique, in which it is assumed that the relationship between E and i is linear (i.e. resistance is a scalar value) in a narrow range (+/−5 mV) around E_corr_. The potential is scanned from -5 mV vs. E_corr_ to +5 mV vs. E_corr_ at specified intervals (e.g. 1 mV) and scan rate (e.g. 1 mV/sec). The selection of these measurement parameters is dependent upon the electrolyte/metal system being examined (Mansfeld 1976, Scully 2000). The slope of the curve provides R_p_'. The inverse of R_p_ (R_p_^−1^) is proportional to the corrosion rate i_corr_. This approach is used in field tests and forms the basis of commercial corrosion rate monitors (ASTM G96-90, 2018).

Applications of R_p_ techniques have been reported by King et al. (1986) in a study of the corrosion behavior of iron pipes in environments containing SRB. In a similar study, Kasahara and Kajiyama (1986) used R_p_ measurements with a compensation of R_s_ and reported results for active and inactive SRB. Lee et al. (2004) used linear polarization measurements to demonstrate that corrosion of CS was more aggressive in stagnant anaerobic seawater than in stagnant aerobic seawater over a 396-day exposure. In general, instantaneous corrosion rates for the anaerobic condition were two orders of magnitude higher than the aerobic condition.

Significant errors in the calculation of corrosion rates can occur for electrolytes of low conductivity or systems with very high corrosion rates (low R_p_) if a correction for R_s_ is not applied. Corrosion rates will be underestimated in these cases. Additional problems can arise from the effects of the sweep rate used to determine R_p_ according to equation (1). If the sweep rate is too high, the experimental value of R_p_ will be too low, and the calculated corrosion rate will be too high. For localized corrosion, experimental R_p_ data should be used as a qualitative indication that rapid corrosion is occurring. Large fluctuations of R_p_ with time are often observed for systems undergoing pitting or crevice corrosion. R_p_ data are meaningful for general or uniform corrosion but less so for localized corrosion, including MIC. Additionally, the use of Stern-Geary theory, where corrosion rate is inversely proportional to R_p_ at potentials close to E_corr_, is valid for conditions controlled by electron transfer but not for diffusion-controlled systems as frequently found in MIC. R_p_ and E_corr_ techniques are often performed simultaneously during monitoring as the two techniques provide complementary EC data. Measurement of the time-dependent R_p_/E_corr_ trend is one of the most commonly used corrosion monitoring techniques in field conditions.

### Electrochemical impedance spectroscopy (EIS)

EIS techniques record impedance data as a function of the frequency of an applied signal at a fixed potential. For comparison, EIS is an AC (alternating current) frequency-dependent technique compared with the DC (direct current) polarization technique described above. A large frequency range (mHz to kHz) must be investigated to obtain a complete impedance spectrum. Dowling et al. (1988) and Franklin et al. (1991) demonstrated that the small signals required for EIS do not adversely affect the numbers, viability, and activity of microorganisms within a biofilm. EIS data may be used to determine R_p_, the inverse of corrosion rate. EIS is commonly used for steady-state conditions (uniform corrosion); however, sophisticated models have been developed for localized corrosion (Mansfeld et al. 1982, Kendig et al. 1983).

Several reports have been published in which EIS has been used to study the role of SRB in the corrosion of buried pipes (Kasahara and Kajiyama 1986, King et al. 1986, 1991). Formation of biofilms and calcareous deposits on three SSs and titanium during exposure to natural seawater was monitored using EIS and surface analysis (Mansfeld et al. 1990). Dowling et al. (1988) used EIS to study the corrosion behavior of CSs affected by bacteria and attempted to determine R_p_ from the EIS data.

EIS is also useful for studying the MIC of metals with protective coatings. Jones-Meehan et al. (1991) used EIS to determine the effects of several mixed microbiological communities on the protective properties of epoxy top coatings over zinc-primed steel. Spectra for the control remained capacitive, indicating intact coatings, while spectra for five of six samples exposed to mixed cultures of bacteria indicated corrosion and delamination.

While EIS can provide useful information for MIC studies, it potentially requires an increased level of understanding compared to some of the other EC methods in order to be able to correctly interpret the results. One of the key issues is the determination of the equivalent electrical circuits used for modeling of the solid/electrolyte interface.

### Medium and large signal polarization

Medium and large signal polarization techniques require potential scans ranging from several tens of mV to several V (see Fig. 6), with the exact range applied depending on the information that the test is aiming to obtain. An external signal is applied to obtain potentiostatic or potentiodynamic polarization curves as well as pitting scans. Medium signal polarization is often capped at +/−200 mV vs. E_corr_. Medium signal polarization curves can be used to determine i_corr_ by Tafel extrapolation as well as specific electron transfer reactions. With large signal polarization, mass transport-related phenomena can be evaluated based on the limiting current density (i_L_). For metal/electrolyte systems for which an active-passive transition occurs, the passive properties can be evaluated based on the passive current density (i_pass_). Pitting scans are used to determine (E_pit_).

A disadvantage of large-signal polarizations is the destructive nature, i.e. the irreversible changes of surface properties due to the application of large anodic or cathodic potentials. Choice of scan rate is important in MIC studies to reduce effects on biofilm structure and character. The faster the scan rate, the less the impact on microbial activities. Recording polarization curves provides an overview of reactions for a given corrosion system, e.g. charge transfer or diffusion-controlled reactions, passivity, transpassivity, and localized corrosion phenomena. Due to the irreversible changes to biological and chemical surface characteristics, large-scale polarization experimental design should include enough samples for separate anodic and cathodic polarization scans, i.e. larger-scale polarization should not be applied to one sample through the full cathodic and anodic potential ranges. Due to the destructive nature of large-signal polarizations (e.g. inducing pitting), care also needs to be taken not to confuse such changes to the surface of a test sample as being due to corrosion that has taken place prior to the EC testing.

Numerous investigators have used polarization curves to determine the effects of microorganisms on the EC properties of metal surfaces and the resulting corrosion behavior. In most of these studies, comparisons have been made between polarization curves in sterile media with those obtained in the presence of bacteria and fungi. Keresztes et al. (1998) used measurements of E_corr_, R_p_, and potentiostatic polarization measurements to obtain corrosion rates. Culture media containing sulfide of both biogenic and chemical origin were used to determine the effects of metal-sulfide layers. Biocides were used to inhibit bacterial metabolic activity. An atomic force microscope was used to image the topography of sulfide layers. They concluded that SRB produced continuous and localized sulfide, regenerating anodic sites and—in the case of iron—activating cathodic sites in the vicinity of the anodes.

In general, EC techniques are valuable methods for mechanistic investigations and monitoring of MIC. Like all specialized techniques, selection, application, and data interpretation for each EC technique are the key learning curve for any scientist wishing to utilize them. The most common mistake researchers make using these techniques is overinterpretation of data. In addition, all of the described techniques are highly dependent upon the specific environment of application, as well as changes in the environment (e.g. temperature, pressure, and flow rate) over time. For example, temperature fluctuations can directly affect EC behavior. Finally, as MIC is a wide interdisciplinary phenomenon, EC should be just one part of the overall experimental design in addition to other chemical and biological measurement techniques.

## Future perspectives

MIC research is a truly interdisciplinary field requiring various expertise. In addition to basic laboratory skills, microbiologists have a wealth of advanced knowledge and expertise that could be applied to help solve some of the key issues in this important topic. For example, microbial ecology studies can provide important nutrient utilization rate data required for predictive model development (Okabe and Characklis 1992). Modeling ecological networks and functional interactions of community members of the microbial consortia involved in corrosion may further our understanding of key players in and factors influencing MIC. Evolutionary studies can offer important information on the differences between field isolates and culture collection species typically used in laboratory studies. Work could be performed to produce targeted detection that finds specific gene markers related to corrosion rather than just general phenotypes (e.g. sulfate reduction), genetic, or metabolite markers that may serve as leading indicators of MIC for helping to optimize treatments or developing new mitigation strategies. The transfer of knowledge on and the application of the latest analytical methods (e.g. microscopy, isolation, species identification/sequencing, metabolomics, transcriptomics, proteomics, etc.) to MIC research are likely to provide further useful insights. Microbiologists are also needed to provide insights/knowledge to the development of laboratory and field-testing standards and best-practice guides relevant to MIC (e.g. field testing, biocide selection, sample handling/preservation for genomics analysis, etc.).

As discussed throughout this review, reliable diagnosis of MIC requires MLOE, where in general, an increased number of measurements of different types (e.g. microbiological, chemical, metallurgical) improves confidence in the conclusions that can be drawn. There are at present, however, no guidelines as to how many types of measurements are needed or if specific measurements are better than others. While efforts on this are underway (e.g. in updated NACE/AMPP and other standards), it is an area that could definitely benefit from additional work and collaborations that have the potential for major impact.

Close collaboration among disciplines involved in MIC research is truly the key to efficiently tackling current challenges. The association for material protection and performance (AMPP), the international biodeterioration and biodegradation society (IBBS), and the EUROCORR, among others, aim to bring together representatives of various fields as well as provide platforms for continuous communication between industry and academy. A frequent exchange between various societies would be also beneficial for the understanding of MIC.

There are numerous challenges that can affect interdisciplinary collaboration in MIC research and its application in industry such as differences in research priorities, communication barriers, funding constraints, and even the format of guidelines (Wade et al. 2023). However, as we have seen in the oil and gas industry (geno-MIC Project), new developments stem from close cooperation among industrial operators and stakeholders as well as researchers from academia. Such collaborations can greatly improve our understanding of MIC mechanisms, develop new monitoring techniques and green mitigation measures. The Euro-MIC COST Action (euro-mic.org), launched in the fall of 2021, has similar aims, involving MIC researchers across the globe from various disciplines as well as stakeholders from various industrial sectors. Many of these groups/organizations are open to new members, are a great way to develop one’s knowledge of other disciplines and make further contacts with experts in the field of MIC.

International networks encourage debate on urgent global challenges, as well as international collaborations, particularly within the Higher Education Sector, allowing graduates and early career researchers to acquire knowledge in a diverse and professional environment, as well as new perspectives on their research, preventing stigmas and paradigms and promoting unity of efforts by bringing together stakeholders with specialized and complementary expertise to address critical industry-led scientific challenges, and enhancing educational and research outcomes.

Corrosion training and education programs available to date are often limited to a specific area without giving a broader overview of MIC. The cooperation of different panels, knowledge exchange between different disciplines as well as between different generations of scientists needs to be enhanced, and MIC training and education programs should be developed. Dissemination is key to quickly and efficiently deal with MIC issues.

## Concluding remarks

The paper has provided an overview of some of the key aspects of MIC to provide background to microbiologists, as well as those new to the field, with a focus on non-microbiological aspects of MIC. A major aim of the work is to help break down some of the siloed knowledge and research being undertaken on this topic and encourage multidisciplinary collaborations.

Some of the key messages from the paper include:

- MIC does not describe a single mechanism for corrosion, and there is still much more work to do to clarify some of the mechanisms involved.
- Correct diagnosis of MIC requires MLOE (microbiological, metallurgical, and chemical), as well as information on engineering design and operations.
- A wide range of materials can suffer degradation due to MIC, and various examples have been discussed with an emphasis on metal alloys, including methods for analyzing metallurgical aspects of MIC.
- Models for studying MIC vary from single strain through to real-world consortia with each type allowing different aspects to be studied. There is significant potential for developing and testing models that more accurately mimic the ‘real world’.
- Management of MIC involves the key aspects of threat assessment, mitigation/prevention and monitoring. Again, many of these require MLOE to provide accurate and useful information.
- EC techniques can provide critical information for MIC studies in the laboratory and in the field; however, there are numerous limitations, so care needs to be taken when designing, performing, and interpreting results from these methods.

MIC is a field growing at an exponential rate, with huge potential for new scientific discoveries. MIC researchers and specialists with multidisciplinary background are critical to drive this field forward. Microbiologists with an interdisciplinary mindset will have an important role in shaping the future of MIC research.
